# Clinical course of severe congenital aortic valve stenosis in children

**DOI:** 10.1016/j.ijcchd.2025.100626

**Published:** 2025-10-09

**Authors:** Maximiliaan L. Notenboom, Sencer Albayrak, Kevin M. Veen, Ingrid van Beynum, Rebecca Swens, Jolien W. Roos-Hesselink, Pieter C. van de Woestijne, Jonathan R.G. Etnel, Willem A. Helbing, Johanna J.M. Takkenberg, Ad J.J.C. Bogers

**Affiliations:** a Department of Cardiothoracic Surgery, Erasmus MC, Rotterdam, the Netherlands; b Department of Pediatrics, Division of Pediatric Cardiology, Erasmus MC-Sophia Children's Hospital, Rotterdam, the Netherlands; c Department of Cardiology, Erasmus MC, Rotterdam, the Netherlands

**Keywords:** Aortic valve, Aortic valve stenosis, Congenital heart disease, Natural course, Pediatrics, Decision-making

## Abstract

**Background:**

Congenital valvular aortic stenosis (VAS) in children requires lifelong follow-up. An overview of age-specific treatment pathways from first diagnosis of severe VAS is lacking.

**Objectives:**

To assess patient journeys and outcomes after severe pediatric VAS diagnosis, by describing its clinical course and treatment trajectories based on a 37-year single-center experience.

**Methods:**

Baseline and time-related data of children diagnosed with severe congenital VAS between 1985 and 2022 were retrospectively collected. Time-related death (Kaplan-Meier estimator) and intervention occurrence (Aalen-Johansen estimator) were analyzed.

**Results:**

245 children (73.1 % male, median age: 1.2 years (IQR:0.1–7.0)) were diagnosed with severe VAS (53 aged <30 days, 74 between 30d-1y, 84 between 1y-12y, 34 between 12y-18y). Median survival follow-up was 23.3 years (IQR:10.3–31.2) (99.0 % complete). Thirty-five-year incidence of death after diagnosis was 16.2 %(95 %CI:9.7–22.2 %). Of 245 patients, 211 patients (86.1 %) underwent an intervention and 34 (13.9 %) did not undergo an intervention. Of these 34 children, 7 children showed time-related Doppler gradient regression to mild-or-moderate VAS and 17 had stable severe VAS. These 24 children experienced a 6.0 % incidence of death at 30-years after diagnosis. The most common intervention over time (47.5 %) was balloon valvuloplasty, especially in neonates and infants, followed by aortic valve replacement (37.5 %), especially in older children.

**Conclusions:**

This study highlights the vast heterogeneity of treatment pathways and outcomes in children diagnosed with severe VAS at different ages. The observed stabilization of severe VAS or regression of the serial peak Doppler gradient to mild-or-moderate VAS without symptoms in 24 children highlights the need for better insight into determinants of disease course.

## Graphical abstract

**Severe congenital valvular aortic stenosis in children: a single-center experience**. An overview of treatment pathways in children diagnosed with severe VAS by Doppler echocardiography. Sankey plots allow for visualization of sequential decisions over time. The thickness of the line corresponds to the proportion of patients receiving that treatment. The first bar indicates the time of diagnosis (100 %). The second bar represents the first intervention, the third bar the second, etcetera.

**Figure:**
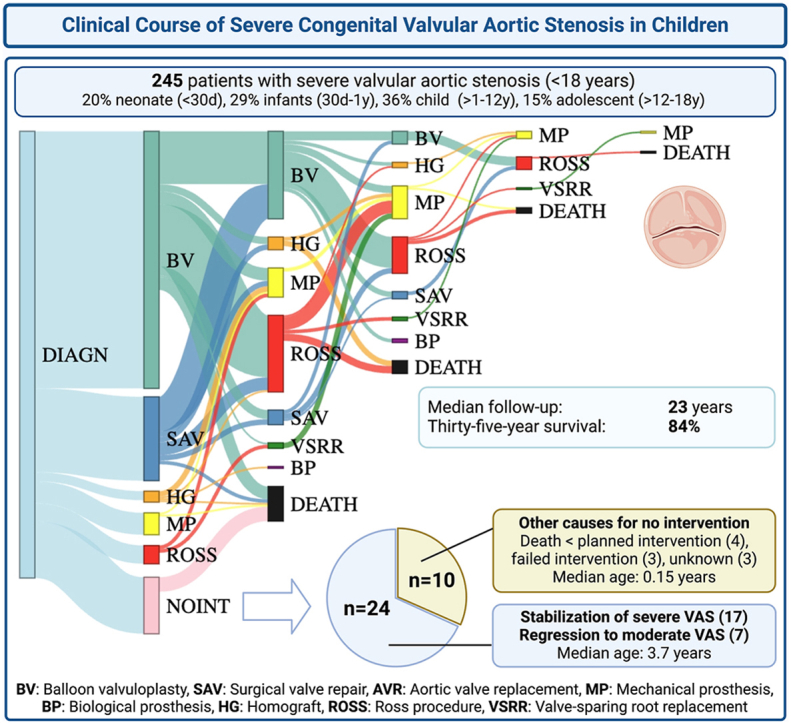

## Abbreviations

AoV: Aortic valve
AVR: Aortic valve replacement
BAV: Bicuspid aortic valve
BP-AVR: Bioprosthetic aortic valve replacement
BV: Balloon aortic valvuloplasty
HG-AVR: Homograft aortic valve replacement
LV: Left ventricle/left ventricular
MP-AVR: Mechanical aortic valve replacement
MV: Mitral valve
VAS: Valvular aortic stenosis
VSRR: Valve-sparing aortic root replacement

## 1 Introduction

In pediatric valvular aortic stenosis (VAS), the severity of the stenosis, impairment of left ventricular (LV) function and presence of concomitant anomalies determine the clinical presentation, ranging from mild stenosis that becomes noticeably in adolescence or after childhood, to severe, life-threatening, stenosis in the first months of life, requiring immediate intervention [1]. Most children diagnosed with severe VAS will require at least one intervention throughout their lifespan and often more interventions are needed [2].

Available treatment options for congenital VAS include watchful waiting, balloon aortic valvuloplasty (BV), surgical aortic valve (AoV) repair and AoV replacement (AVR). Each treatment option in the management of VAS in children is associated with unique benefits and drawbacks [1,3,4]. Therefore, strategic planning of these interventions over the course of the lives of these patients is of utmost importance.

A comprehensive overview of the age-specific clinical course, outcomes and treatment pathways of children with severe congenital VAS is lacking. Therefore, the aim of this study is to provide an overview of sequential treatment pathways and associated long-term outcomes of children diagnosed with severe congenital VAS in our center between 1985 and 2022.

## 2 Methods

### 2.1 Patients

All patients diagnosed with severe VAS <18 years at the Erasmus University Medical Center between January 1985 and February 2022 were included. Severe VAS was defined as valvular stenosis with a peak transvalvular gradient (PG) ≥64 mmHg measured at rest by either Doppler echocardiography (maximum transvalvular velocity (AV Vmax) ≥4 m/s^5^) or cardiac catheterization (peak-to-peak gradient), according to the guidelines [6]. For diagnosis of severe VAS, children required one measurement of PG ≥ 64 mmHg followed by intervention, or two successive PG measurements ≥64 mmHg. The individual echocardiograms and clinical context in patients who did not undergo an intervention were carefully revised by a dedicated pediatric echocardiographer to confirm diagnosis. Patients with any event that could increase cardiac output, e.g., crying, resistance, fever, during diagnosis that was not followed by an intervention, were excluded.

Cases of critical VAS requiring immediate intervention on clinical grounds, including those with low-flow, low-gradient (LF-LG) VAS with LV dysfunction, were included, which was the only rule of exception for the ≥64 mmHg AoV PG threshold. Patients with only or primarily sub- and/or supravalvular stenosis without a primary valvular component were excluded, as were children with hypoplastic left heart syndrome determined by multidisciplinary discussion.

### 2.2 Data and definitions

For this cohort study, the STROBE guidelines (Strengthening the Reporting of Observational Studies in Epidemiology) and checklist were followed (Supplement 1) [7].

Patients were identified through an electronic database search including diagnosis codes, intervention codes and text keywords. Data were collected retrospectively. Clinical outcomes were defined according to the guidelines for reporting mortality and morbidity after heart valve interventions, described by Akins [8]. Early events were defined as events occurring ≤30 days after intervention/diagnosis. Late events were defined as events occurring >30 days after intervention/diagnosis. Interventions were divided into valve-related and other, non-AoV-related cardiovascular interventions (e.g. on coarctation). Time-related clinical visits, imaging studies and interventions were collected as repeated measurements. Survival status of all patients was retrieved on November 17, 2023, from the Dutch personal records database. For outcomes other than death, the censoring date was defined as the latest clinical follow-up. To calculate follow-up completeness, the modified Clark's C method was used, with end-of-study date defined as the date of database closure minus one year (December 29, 2021).

Maximum velocities across the AoV (prosthesis) were obtained by Doppler measurement and PG was calculated by the modified Bernoulli equation [5].

For ages ≤18 years, the modified age-specific Ross Classification for heart failure in children was used to assess heart failure severity [9]. For ages ≥18 years, the New York Heart Association classification was used. Electronic patient files were screened and the classification at diagnosis and visits was graded based on symptomatology (Supplement 2).

### 2.3 Statistical analysis

Continuous data are presented as means ± standard deviation (Gaussian distribution) or as medians with interquartile range (IQR) (non-Gaussian distribution). Normality was tested using the Shapiro-Wilk test. Categorical data are presented as frequencies with percentage. Comparisons among continuous data were made with the student's t-test or the Mann–Whitney *U* test, as appropriate. Comparisons of categorical data were made with the χ2 test or the Fisher's exact test, as appropriate.

Statistical analyses were stratified according to age at diagnosis of severe VAS: neonates (<30 days), infants (30 days–1 year), children (>1 year–12 years) and adolescents (>12 years–18 years). The Kaplan-Meier estimator was used to estimate late survival, presented as incidence of death. Comparisons between age-specific Kaplan-Meier estimates were performed through the log-rank test. Cumulative incidence of AoV (re)intervention was assessed in presence of death as a competing risk through the Aalen-Johansen estimator to generate multi-state curves. Gray's test was used to evaluate hypotheses of equality of cause-specific cumulative incidence functions between groups. The observed incidence of death was compared to the matched-general-population. Mortality rates from the Dutch population were obtained from the Human Mortality Database. The estimation of matched-general-population mortality involved utilizing individual-level parameters, including sex, country, age, calendar year and censoring time [10].

To handle missing baseline data, multiple imputation by chained equations using the statistical mice package in R was performed (Supplement 3). The proportion of missing data, along with their correlations, are provided in Supplement 4 and 5.

Uni- and multivariable Cox proportional hazards models were employed to identify risk factors associated with death and time-to-first-intervention. Model selection for multivariable Cox models and mixed-effects model methodology for echocardiographic analyses is described in Supplement 6. All statistical analyses were performed using R version 4.0.2. P-values of <0.05 were considered statistically significant.

### 2.4 Recurrent events

Sequential treatment pathways were visualized, stratified by age and era (1985–2003 vs 2004–2022). To model recurring interventions during follow-up, the cumulative mean number of interventions was computed and visualized using a non-parametric, mean cumulative function.

### 2.5 Ethics statement

The protocol for this study was approved by the local medical ethics committee of the Erasmus MC (MEC-2014-244). The need for individual informed consent was waived.

## 3 Results

After screening electronic patient records, 245 children were diagnosed with severe congenital VAS and included (Supplement 7). The median age at diagnosis was 1.2 years (IQR:0.1–7.0 years) and 73.1 % were male.

Median survival follow-up was 22.6 years (IQR:9.7–30.5) and 99 % complete. Patients had a median number of 14 outpatient visits (IQR:8–22). Median echocardiographic follow-up was 14.7 years (IQR:4.9–26.5).

### 3.1 Patients

**Fig. 1:**
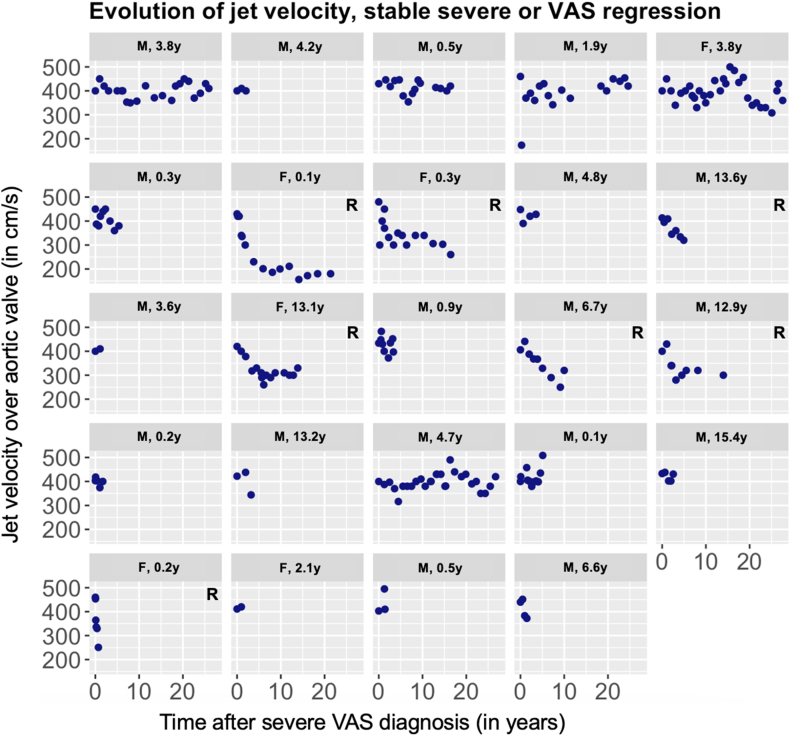
Serial Doppler jet velocities for the 24 children with a decrease or stabilization of their gradient without intervention. The Y-axis represents the peak jet velocity (in cm/s) measured over the aortic valve by use of Doppler echocardiography; the X-axis represents time (in years). R = gradient Regression.

Patient characteristics and echocardiographic details at diagnosis are presented in Table 1. For neonatal vs. non-neonatal VAS, baseline characteristics are presented in Supplement 8. One patient had confirmed genetic disease (Turner Syndrome). Baseline characteristics of 211 (86.1 %) patients who underwent an AoV intervention during follow-up, compared to 34 (13.9 %) who did not are presented in Table 2. The reasons for the clinical decision to refrain from intervening were: asymptomatic severe VAS that eventually regressed to moderate/mild on echo Doppler (n = 7), asymptomatic severe VAS with normal LV function (n = 17), death before planned intervention (n = 4), failed/unfeasible intervention (n = 3), and unknown (n = 3). Reasons for not intervening along with detailed patient characteristics are summarized in Supplement 9. The serial Doppler jet velocity measurements of 24 children with VAS gradient regression (n = 7) and stabilization (n = 17) are plotted in Fig. 1.

**Table 1:** Patient characteristics at time of diagnosis of severe congenital VAS, stratified by age at diagnosis.

	All	Neonates (<30d)	Infants (30d-1y)	Children (1y-12y)	Adolescent (12-18y)	P-value	Missing
**Variable**	n = 245 (100 %)	n = 53 (21.6 %)	n = 74 (30.2 %)	n = 84 (34.3 %)	n = 34 (13.9 %)		**%**
Age, y	0.94 [0.10, 6.23]	0.01 [0.00, 0.05]	0.22 [0.14, 0.35]	4.77 [2.97, 6.99]	14.59 [13.46, 15.99]	**<0.001∗**	0.0
Male	177 (72.2)	33 (62.3)	56 (75.7)	59 (70.2)	29 (85.3)	0.106	0.0
Weight, kg	8.50 [4.20, 21.80]	3.38 [2.94, 3.85]	5.46 [4.46, 6.42]	18.60 [14.00, 24.00]	57.00 [47.00, 64.00]	**<0.001∗**	8.2
Height, cm	80.5 [56.6, 120.6]	50.5 [48.5, 52.0]	60.0 [56.8, 64.3]	108.5 [94.0, 121.0]	168.0 [160.0, 174.4]	**<0.001∗**	18.4
BSA, m^2^	0.48 [0.27, 0.88]	0.21 [0.19, 0.23]	0.29 [0.27, 0.33]	0.74 [0.59, 0.90]	1.65 [1.46, 1.73]	**<0.001∗**	18.8
Aortic valve morphology^a^						**0.020∗∗**	16.7
Unicuspid	10 (4.9)	7 (17.1)	2 (3.1)	1 (1.4)	0 (0.0)	**0.002∗∗**	
Bicuspid	152 (74.5)	28 (68.3)	51 (78.5)	54 (77.1)	19 (67.9)	0.517	
Tricuspid	42 (20.6)	6 (14.6)	12 (18.5)	15 (21.4)	9 (32.1)	0.336	
Ross functional classification						**<0.001∗∗∗**	9.0
Ross I	152 (68.2)	15 (34.1)	46 (66.7)	65 (84.4)	26 (78.8)	**<0.001∗∗∗**	
Ross II	28 (12.6)	6 (13.6)	7 (10.1)	9 (11.7)	6 (18.2)	0.702	
Ross III	13 (5.8)	2 (4.5)	7 (10.1)	3 (3.9)	1 (3.0)	0.427	
Ross IV	30 (13.5)	21 (47.7)	9 (13.0)	0 (0.0)	0 (0.0)	**<0.001∗∗∗**	
Concomitant anomalies^b^	73 (29.8)	31 (58.5)	18 (24.3)	20 (23.8)	4 (11.8)	**<0.001∗∗**	11.8
EFE	18 (7.3)	17 (32.1)	1 (1.4)	0 (0.0)	0 (0.0)	**<0.001∗∗**	0.0
Arch anomalies	5 (2.0)	3 (5.7)	0 (0.0)	2 (2.4)	0 (0.0)	0.123	
COA	29 (11.8)	6 (11.3)	9 (12.2)	13 (15.5)	1 (2.9)	0.288	0.0
VSD	13 (5.3)	2 (3.8)	3 (4.1)	6 (7.1)	2 (5.9)	0.798	0.0
MV dysfunction (grade≥3)	25 (10.2)	11 (20.8)	9 (12.2)	4 (4.8)	1 (2.9)	**0.011∗∗∗**	0.0
PV dysfunction (grade≥3)	4 (1.6)	1 (1.9)	1 (1.4)	1 (1.2)	1 (2.9)	0.825	0.0
Ventilator	7 (3.0)	7 (13.7)	0 (0.0)	0 (0.0)	0 (0.0)	**<0.001∗∗**	4.9
Diuretics	12 (5.2)	5 (9.8)	7 (9.9)	0 (0.0)	0 (0.0)	**0.010∗∗∗**	5.3
Inotropics	9 (3.9)	6 (11.8)	3 (4.2)	0 (0.0)	0 (0.0)	**0.005∗∗**	5.3
Subaortic stenosis	19 (7.8)	2 (3.8)	4 (5.4)	13 (15.5)	0 (0.0)	**0.012∗∗∗∗**	0.0
Aortic valve Vmax, m/s	4.34 [4.03, 4.81]	4.19 [3.40, 4.65]	4.50 [4.18, 5.00]	4.33 [4.04, 4.85]	4.30 [4.07, 4.49]	**0.004∗∗**	6.5
Critical VAS	18 (7.3)	13 (24.5)	4 (5.4)	1 (1.2)	0 (0.0)	**<0.001∗∗**	0.0
Vmax below 4.0 m/s	20 (8.7)	17 (39.5)	1 (1.4)	2 (2.4)	0 (0.0)	**<0.001∗∗**	6.5
Vmax above 5.0 m/s	53 (23.1)	8 (18.6)	20 (29.0)	20 (24.1)	5 (14.7)	0.358	6.5

**Legend**: *Values are expressed as n (%) or as median [IQR], unless stated otherwise.*

∗ P-value significant across all four groups.

∗∗ P-value significant for Neonatal (n = 53) versus all other groups.

∗∗∗ P-value significant for both Neonatal (n = 53) and Infant (n = 74) versus Children (n = 84) and Adolescents (n = 34).

∗∗∗∗ P-value significant for Children (n = 84) versus all other groups.

a Other patients: valve morphology was unknown.

b Concomitant anomalies may coexist or overlap with each other and consist of Interrupted aortic arch, Coarctation of the aorta, Ventricular septal defect, Atrial septal defect, Patent foramen ovale, Patent ductus arteriosus, Mitral valve stenosis/insufficiency (grade≥3), Pulmonary valve stenosis/insufficiency (grade≥3), Tricuspid valve regurgitation (grade≥3) and Subvalvular aortic stenosis.^e^ AV Vmax < 4 m/s because patient had LF-LG severe valvular AS and therefore still included. Abbreviations, AV: Aortic valve; BSA = Body Surface Area; FS: Fractional Shortening. **No corrections for multiple testing were applied**.

**Table 2:** Patient characteristics at diagnosis for patients with aortic valve intervention versus those without an intervention.

	Intervention group n = 211	No intervention group n = 34			**P-Value**	**Missing** (%)
	**AoV intervention** n = 211	**Gradient regression** n = 7	**Gradient stabilization** n = 17	**Other causes** n = 10		
Age, y	0.93 [0.10, 6.58]	6.68 [0.93, 9.25]	3.62 [0.51, 4.71]	0.15 [0.01, 1.45]	0.059	0.0
Male	151 (71.6)	3 (42.9)	15 (88.2)	18 (75.0)	0.137	0.0
Weight, kg	8.30 [4.12, 21.73]	20.0 [5.55, 53.35]	16.25 [12.29, 22.75]	4.38 [3.75, 6.40]	**0.017∗**	8.2
Height, cm	79.90 [56.35, 121.00]	121.0 [58.5, 164.1]	100.5 [75.8, 113.6]	54.75 [52.62, 63.25]	0.117	18.4
BSA, m^2^	0.48 [0.28, 0.88]	0.83 [0.28, 1.57]	0.65 [0.54, 0.80]	0.23 [0.22, 0.31]	0.064	18.8
Age groups					0.211	0.0
Neonates (<30d)	47 (22.3)	1 (14.3)	1 (5.9)	4 (40.0)		
Infants (30d-1y)	64 (30.3)	2 (28.6)	5 (29.4)	3 (30.0)		
Children (1y-12y)	71 (33.6)	1 (14.3)	9 (52.9)	3 (30.0)		
Adolescents (12y-18y)	29 (13.7)	3 (42.9)	2 (11.8)	0 (0.0)		
AV cusp morphology					0.380	16.7
Unicuspid	9 (5.0)	0 (0.0)	0 (0.0)	1 (25.0)		
Bicuspid	130 (72.6)	7 (100.0)	12 (85.7)	3 (75.0)		
Tricuspid	40 (22.3)	0 (0.0)	2 (14.3)	0 (0.0)		
Ross Classification						9.0
Ross I	128 (66.0)	7 (100.0)	14 (93.3)	3 (42.9)	**0.017∗∗∗**	
Ross II	26 (13.4)	0 (0.0)	1 (6.7)	1 (14.3)	0.654	
Ross III	13 (6.7)	0 (0.0)	0 (0.0)	0 (0.0)	0.559	
Ross IV	27 (13.9)	0 (0.0)	0 (0.0)	3 (42.9)	**0.034∗∗∗∗**	
Concomitant anomalies^a^	62 (29.4)	1 (14.3)	4 (23.5)	6 (60.0)	0.138	11.8
EFE	16 (7.6)	0 (0.0)	0 (0.0)	2 (20.0)		
VSD	9 (4.3)	1 (14.3)	2 (11.8)	1 (10.0)		
Arch abnormalities	3 (1.4)	0 (0.0)	1 (5.9)	1 (10.0)		
Coarctation	25 (11.8)	1 (14.3)	3 (17.6)	0 (0.0)		
MV dysfunction (grade≥3)	23 (10.9)	0 (0.0)	0 (0.0)	2 (20.0)		
Ventilator	7 (3.5)	0 (0.0)	0 (0.0)	0 (0.0)	0.755	4.9
Diuretics	12 (6.0)	0 (0.0)	0 (0.0)	0 (0.0)	0.552	5.3
Inotropics	9 (4.5)	0 (0.0)	0 (0.0)	0 (0.0)	0.670	5.3
Subaortic stenosis	15 (8.0)	0 (0.0)	1 (5.9)	3 (42.9)	0.089	0.0
AV Vmax, m/s	4.40 [4.07, 5.00]	4.20 [4.06, 4.45]	4.03 [4.00, 4.34]	4.74 [4.47, 5.00]	**0.043∗∗**	6.5
Critical VAS	17 (8.1)	0 (0.0)	0 (0.0)	1 (10.0)	0.539	0.0

**Legend**: *Values are expressed as n (%) or as median [IQR], unless stated otherwise.*

∗ P-value significant for Stabilization cohort (n = 17) versus all other cohorts.

∗∗ P-value significant for Stabilization cohort (n = 17) versus Other causes cohort (n = 10).

∗∗∗ P-value significant for Regression cohort (n = 7) and Stabilization cohort (n = 17) versus Other causes cohort (n = 10).

∗∗∗∗ P-value significant for Regression cohort (n = 7) and Stabilization cohort (n = 17) versus all other groups.

a Kruskal-Wallis.

b χ2 test.

c Fisher's exact test.

a Concomitant anomalies may coexist or overlap with each other and consist of Interrupted aortic arch, Coarctation of the aorta, Ventricular septal defect, Atrial septal defect, Patent foramen ovale, Patent ductus arteriosus, Mitral valve stenosis/insufficiency (≥moderate), Pulmonary valve stenosis/insufficiency (≥moderate), Tricuspid valve regurgitation (≥moderate) and Subvalvular aortic stenosis. Abbreviations, AV: Aortic valve; BSA = Body Surface Area; FS: Fractional shortening. **No corrections for multiple testing were applied**.

The mean echo follow-up in patients with VAS regression/stabilization (n = 24) was 10.2 years (median: 5.0, IQR:1.8–19.6 years).

### 3.2 Survival

Of all 245 children, 30 (12.2 %) patients died during follow-up (age at diagnosis: 15 neonates, six infants, eight children, one adolescent). Of all 30 deaths, 9 (30.0 %) occurred within 30 days after diagnosis. Nineteen deaths (63.3 %) were cardiac, three (10.0 %) non-cardiac, and in eight (26.7 %) the cause of death was unknown. Cardiac deaths (n = 19) consisted of 7 deaths early after diagnosis (6 neonates, 1 infant), 9 valve-related deaths (6 valve-event-related deaths and 3 sudden, unexplained deaths) and 3 non-valve-related cardiac deaths (one complete heart block, two heart failure with normal AoV function). Thirty-day mortality after an intervention was observed in seven patients, following four BVs (three neonates and one 5-year-old), one AoV repair in a neonate, one Ross procedure in an infant and one HG-AVR in a 12-year-old. Thirty-day mortality risk after BV was 2.1 %, after AoV repair 1.8 %, after Ross procedure 1.3 % and after HG-AVR 6.7 %; p-value = 0.588).

**Fig. 2:**
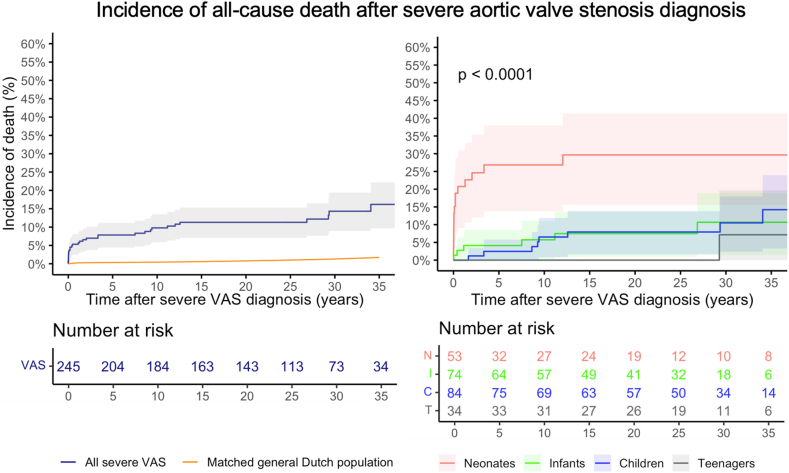
Cumulative incidence of all-cause death after diagnosis of severe VAS. Incidence of death for all children compared to death incidence in the matched-general-Dutch-population (left), and stratified by age at diagnosis (right).

Four (1.5 %) children (three neonates, one infant) died before a planned AoV intervention. A detailed description of these deaths and reasons for failed/unfeasible interventions, are provided in Supplement 10. Thirty-five-year incidence of death after diagnosis of severe VAS was 16.2 % (95 % Confidence Interval (CI):9.7–22.2 %). Death probability in the matched-general-Dutch-population was 1.7 % at 35 years. This translates to a relative patient survival of 85.2 % (95 %CI:70.9–95.7 %) of expected. Fig. 2 represents the cumulative incidence of all-cause mortality. Survival for infants and neonates with and without critical VAS and EFE is depicted in Supplement 11.

Thirty-five-year incidence of death after diagnosis was 29.7 %(95 %CI:15.6–41.4 %) in neonates, 10.7 %(95 %CI:1.6–19.0 %) in infants, 14.2 %(95 %CI:3.3–23.9 %) in children and 7.1 %(95 %CI:0–19.7 %) in adolescents (log-rank p < 0.001).

In patients that did not undergo an AoV intervention(n = 34), survival was as follows: in children that showed peak Doppler gradient regression to moderate/mild VAS (n = 7), 30-year survival was 100 % (median follow-up: 23.6y (IQR:13.6–26.4)). In patients with asymptomatic VAS that remained severe (n = 17), one patient (5.9 %) died two months after diagnosis (median follow-up: 22.0y (IQR:6.3–26.8)). In patients without an intervention due to a failed/unfeasible intervention or unknown reasons (n = 6), three patients (50.0 %) died during a median follow up of 0.4y (IQR:0.03–9.4), in addition to the patients that died before a planned intervention (n = 4).

### 3.3 Intervention details

Median duration of clinical follow-up was 14.6 years (IQR:5.1–26.2, 4198 patient-years). Follow-up completeness for clinical events other than survival was 83 %. In total, 508 interventions were performed in 218 patients, consisting of 402 (79.1 %) aortic-valve-related interventions in 211 patients and 106 (20.9 %) non-valve-related interventions in 67 patients, including 38 coarctation, 22 ventricular septal defect closures, 22 pulmonary valve interventions (post-Ross), 12 aortic arch operations, five mitral valve interventions, one tricuspid valve repair, and 44 other. Table 3 provides the intervention details at the time of first AoV intervention stratified by age. Characteristics of patients during the first, second or third AoV intervention, respectively, are listed in Supplement 12–14.

**Table 3:** Details of aortic valve intervention for patients stratified by age at diagnosis.

Procedures	All 402^a^	106^b^	110^b^	135^b^	51^b^	**P-value∗**
**Patients**	All 211	47 Neonates	64 Infants	71 Children	29 Adolescents	
Number of interventions						0.463
First intervention	211 (52.5)	47 (44.3)	64 (58.2)	71 (52.6)	29 (56.9)	
Second intervention	122 (30.3)	36 (34.0)	30 (27.3)	40 (29.6)	16 (31.4)	
≥Third intervention	69 (17.2)	23 (21.7)	16 (14.5)	24 (17.8)	6 (11.8)	
Time to first intervention, y	0.17 [0.03, 1.17]	0.01 [0.00, 0.03]	0.05 [0.03, 0.27]	0.82 [0.31, 4.73]	0.88 [0.20, 5.06]	**<0.001**
Aortic valve procedures						
BV	191 (47.5)	57 (53.8)	65 (59.1)	58 (43.0)	11 (21.6)	**<0.001**
Surgical AoV repair	55 (13.7)	13 (12.3)	17 (15.5)	21 (15.6)	4 (7.8)	0.504
VSRR^d^	6 (1.5)	2 (1.9)	0 (0.0)	3 (2.2)	1 (2.0)	0.504
AVR	149 (37.0)	34 (32.1)	29 (26.4)	51 (37.8)	35 (68.6)	
Ross procedure	80 (19.9)	26 (24.5)	17 (15.5)	24 (17.8)	13 (25.5)	0.244
MP-AVR	51 (12.7)	4 (3.8)	9 (8.2)	21 (15.6)	17 (33.3)	**<0.001**
BP-AVR	3 (0.7)	1 (0.9)	0 (0.0)	1 (0.7)	1 (2.0)	0.594
HG-AVR	15 (3.7)	3 (2.8)	3 (2.7)	5 (3.7)	4 (7.8)	0.400
Concomitant procedures	47 (11.7)	16 (15.1)	12 (10.9)	18 (13.3)	1 (2.0)	0.099
Arch repair/coarctation repair	17 (4.2)	6 (5.7)	6 (5.5)	5 (3.7)	0 (0.0)	0.349
VSD/ASD/PFO/PDA closure	13 (3.2)	8 (7.5)	3 (2.7)	2 (1.5)	0 (0.0)	**0.024**
Concomitant SAS resection	12 (3.0)	1 (0.9)	1 (0.9)	10 (7.4)	0 (0.0)	**0.003**
Mitral valve surgery	9 (2.2)	3 (2.8)	3 (2.7)	3 (2.2)	0 (0.0)	0.692
(Re-)intervention aetiology						**0.021**
Endocarditis	8 (2.0)	0 (0.0)	2 (1.8)	3 (2.2)	3 (6.0)	
Valve dysfunction post BV	95 (23.9)	34 (32.7)	27 (24.5)	28 (20.9)	6 (12.0)	
Outcomes						
Thirty-day mortality^c^, %	1.7 %	3.8 %	0.9 %	0.7 %	2.0 %	
Total percutaneous^c^ (all), %	2.1 %	5.3 %	0.0 %	1.7 %	0.0 %	
First surgical^c^ (all), %	0.7 %	2.9 %	0.0 %	0.0 %	0.0 %	
Surgical reintervention^c^ (after prior surgical), %	5.6 %	0.0 %	10.0 %	0.0 %	10.0 %	

**Legend:***Values are expressed as n (% of the total number of procedures in that column) or as median [IQR], unless stated otherwise.*

BV: balloon aortic valvuloplasty; SAV: surgical aortic valvotomy; VSRR: valve sparing aortic root replacement; SAS: subvalvular aortic stenosis; AVR: aortic valve replacement; MP-AVR: mechanical aortic valve replacement; BP- AVR: bioprosthetic aortic valve replacement; HG-AVR: homograft aortic valve replacement. **No corrections for multiple testing were applied.**

a Procedures on the aortic valve with or without concomitant procedures.

b P-value applies for comparison of age groups.

c First surgical includes all first surgical interventions, whereas surgical reoperation includes all reinterventions after previous surgery. For thirty day mortality after interventions, age groups correspond to age at death, not at diagnosis.

d VSRR was only performed as a reoperation after prior Ross or BV.

**Fig. 3:**
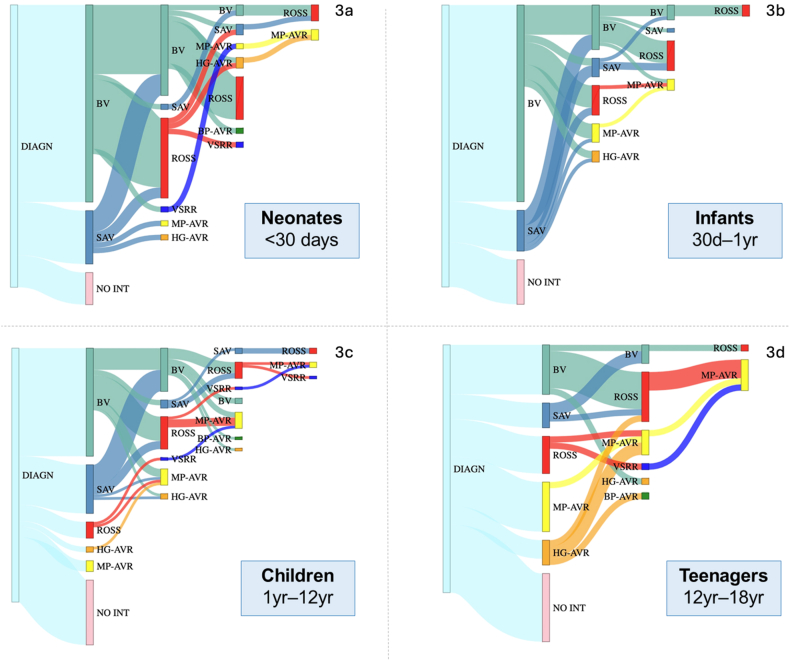
a–d. Sankey plot depicting age-specific sequential treatment pathways from diagnosis. Sankey plots allow for visualization of sequential decisions over time. The thickness of the line corresponds to the proportion of patients receiving that treatment. The first bar indicates the time of diagnosis (100 %). The second bar represents the first intervention, the third bar the second, etcetera. The color of the pathways between the bars represents the latest intervention that the patient is living with. A black bar represents death after an intervention (early/late). Green = BV; Lightblue = SAV/AoV repair; Red = ROSS; Navy = VSRR; Yellow = MP-AVR; Orange = HG-AVR; Darkgreen = BP-AVR. BV=Balloon valvuloplasty; SAV=Surgical AoV repair; VSRR=Valve-sparing root replacement; AVR = Aortic valve replacement; MP = Mechanical prosthesis; HG=Homograft; BP=Bioprosthesis; Black = death.

**Fig. 4:**
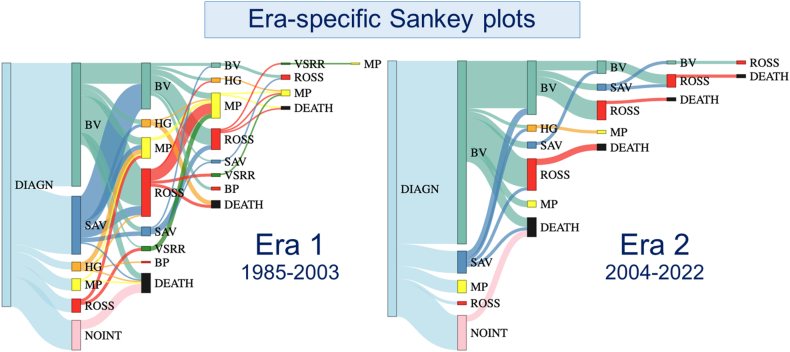
**Sankey plot depicting era-specific sequential treatment pathways (1985**–**2003 vs 2004**–**2022).** See Fig. 3 for a detailed interpretation of the Sankey plot. Green = BV; Lightblue = SAV/AoV repair; Red = ROSS; Navy = VSRR; Yellow = MP-AVR; Orange = HG-AVR; Darkgreen = BP-AVR. BV=Balloon valvuloplasty; SAV=Surgical AoV repair; VSRR=Valve-sparing root replacement; AVR = Aortic valve replacement; MP = Mechanical prosthesis; HG=Homograft; BP=Bioprosthesis; Black = death.

Age-specific AoV-related treatment pathways for patients with an intervention (86.1 %), and those without an AoV intervention (13.9 %) are depicted in Fig. 3a-d, respectively. Era-specific treatment pathways are shown in Fig. 4. In Supplement 15, AoV-treatment pathways for all children are depicted.

### 3.4 Aortic valve (re)intervention

**Fig. 5:**
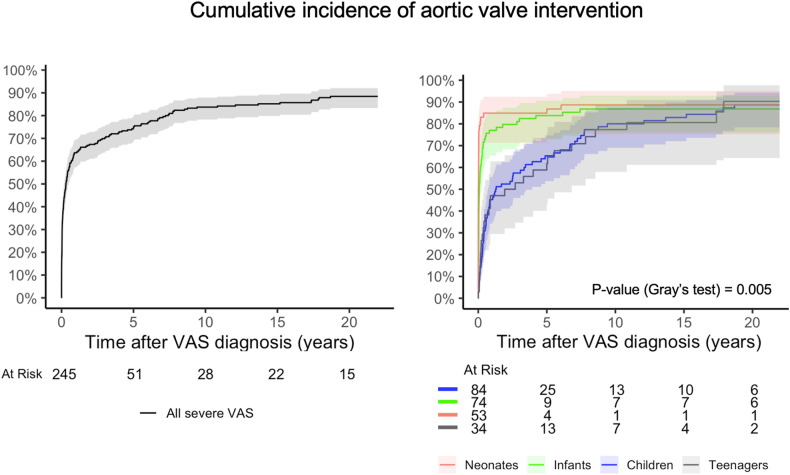
**Cumulative incidence of first aortic valve intervention after diagnosis.** Plot given for all children (left), and for age groups (right). Incidence in presence of death as a competing event.

Cumulative incidence of first AoV intervention was 75.0 % (95 %CI:69.0–80.0 %) at 5 years and 84.0 % (95 %CI:78.0–88.0 %) at 10 years (Fig. 5). For cumulative incidence of first BV and SAV after diagnosis, see Supplement 16–17.

**Fig. 6:**
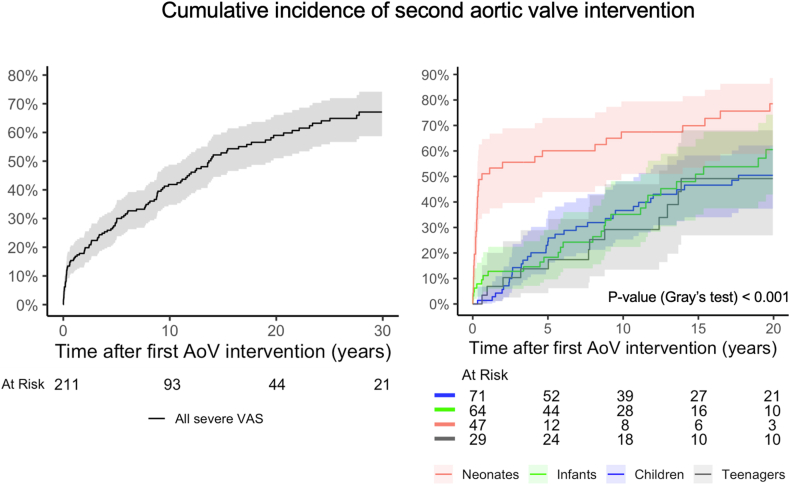
**Cumulative incidence of second aortic valve intervention after first aortic valve intervention**. Plot given for all children (left), and stratified by age at diagnosis (right). Incidence in presence of death as a competing event.

Overall cumulative incidence of an AoV reintervention after first AoV intervention was 29.0 %(95 %CI:23.0–35.0 %) at 5 and 42.0 %(95 %CI:35.0–49.0 %) at 10 years (Fig. 6).

Results of univariable and multivariable Cox proportional hazards regression models for mortality and for time-to-first valve intervention are presented in Table 4.

**Table 4:** Univariable and multivariable Cox regression for mortality and time-to-first intervention.

Mortality	Univariable Cox regression		Multivariable Cox regression^a^	
	HR [95 % CI]	P-value	HR [95 % CI]	P-value
Age group				
Neonates (<30d)	*Reference*	*Reference*		
Infants (30-1y)	0.24 [0.09–0.62]	**0.003**		
Children (>1y-12y)	0.24 [0.10-0.58	**0.001**		
Teenagers (>12y-18y)	0.08 [0.01–0.57]	**0.012**		
AV Vmax, m/s				
Valve morphology				
Unicuspid AoV	9.3 [2.3–37.4]	**0.001**		
Bicuspid AoV	2.1 [0.63–7.1]	0.229		
Tricuspid AoV	*Reference*	*Reference*		
Year of diagnosis	1.06 [1.02–1.09]	**0.001**		
BSA, m2	0.28 [0.10–0.83]	**0.021**		
Fractional shortening	0.97 [0.94–0.99]	**0.008**		
Height, cm	0.99 [0.98–1.00]	**0.015**		
Heart rate	1.02 [1.00–1.03]	**0.005**		
Ross III/IV	4.25 [2.07–8.72]	**<0.001**		
Ventilator, diuretics and/or inotropics	2.27 [0.87–5.95]	0.095		
Subaortic stenosis	2.66 [1.14–6.21]	**0.024**		
Mal	0.63 [0.30–1.33]	0.226		
Weight, kg	0.97 [0.94–1.00]	**0.032**		
Critical VAS	3.16 [1.20–8.30]	**0.018**		
EFE	9.46 [4.2–21.3]	**<0.001**		

**Time to first intervention**				

Age group				
Neonates (<30d)	*Reference*	*Reference*	*Reference*	*Reference*
Infants (30-1y)	0.50 [0.34–0.74]	**<0.001**	0.58 [0.36–0.93]	**0.025**
Children (>1y-12y)	0.23 [0.16–0.34]	**<0.001**	0.32 [0.20–0.51]	**<0.001**
Teenagers (>12y-18y)	0.23 [0.14–0.37]	**<0.001**	0.33 [0.18–0.61]	**<0.001**
AV Vmax, m/s	1.48 [1.15–1.90]	**0.002**	1.94 [1.13–3.35]	**0.023**
Valve morphology				
Unicuspid AoV	*Reference*	*Reference*	*Reference*	*Reference*
Bicuspid AoV	0.27 [0.15–0.50]	**<0.001**	0.45 [0.17–1.18]	0.096
Tricuspid AoV	0.33 [0.17–0.62]	**<0.001**	0.51 [0.19–1.36]	0.165
Year of diagnosis	0.96 [0.95–0.97]	**<0.001**	1.0 [0.98–1.02]	0.876
BSA, m2	0.41 [0.30–0.58]	**<0.001**		
Fractional shortening	0.98 [0.97–0.99]	**0.005**	0.99 [0.97–1.01]	0.173
Height, cm	0.99 [0.98–0.99]	**<0.001**		
Heart rate	1.02 [1.01–1.02]	**<0.001**		
Ross III/IV	3.34 [2.38–4.70]	**<0.001**	1.83 [1.06–3.15]	**0.031**
Ventilator, diuretics and/or inotropics	3.42 [2.18–5.35]	**<0.001**	1.94 [0.75–4.99]	0.152
Subaortic stenosis	1.08 [0.69–1.68]	0.742		
Male	0.90 [0.67–1.21]	0.492		
Weight, kg	0.98 [0.97–0.99]	**<0.001**		
Critical VAS	4.08 [2.44–6.84]	**<0.001**		
EFE	5.01 [2.96–8.48]	**<0.001**	1.97 [0.70–5.55]	0.186

**Legend**: AoV = aortic valve, SAS = subvalvular aortic stenosis, HR = Hazard Ratio, CI = Confidence Interval.

a *Due to correlation with other predictors, BSA, height, heart rate and critical aortic stenosis were omitted in the multivariable Cox proportional hazards models*. ***No corrections for multiple testing were applied*.**

### 3.5 Recurrent AoV interventions

For a general and age-specific overview of recurrent AoV interventions, see Supplement 18–19. In children with an intervention, the median number of interventions was 2 (range: 1–5). There were 211 (86.1 %) patients with ≥1 intervention, 122 (49.8 %) patients with ≥2 interventions, 57 (23.3 %) patients with ≥3 interventions and 12 (4.9 %) patients with ≥4 interventions. In Supplement 20, the mean cumulative function is presented.

### 3.6 Echocardiographic outcome

A total of 4755 echocardiograms were performed. The longitudinal mean evolution of fractional shortening and AoV max jet velocity are presented in Supplement 21, for the regression/stabilization cohort vs. those with intervention (left) and for age groups (right).

## 4 Discussion

This single-center observational study offers insights into treatment paths of children with severe VAS and illustrates patient-tailored intervention strategies and outcomes in our center. The initial and sequential treatment options varied; children ≤1y often underwent BV, whereas AVR was the most common intervention in children >1y. The majority of children diagnosed with severe VAS require multiple and varying types of interventions during a lifetime, particularly neonates. However, at the same time a small proportion of children may not need an intervention, and some show Doppler gradient stabilization or decrease on subsequent measurements without an AoV intervention.

### 4.1 Natural course

In this study, the vast majority of children in all age groups required one or more aortic valve interventions over time, with varying sequential intervention strategies tailored to their individual needs and circumstances. Those children that did not undergo an intervention represent 2 distinct groups: the most severe and the most ‘mild’ phenotypes of severe VAS. Children with the most severe phenotype are often neonates with a low fractional shortening and LF-LG VAS with endocardial fibroelastosis. In these neonates, it appears that early treatment - if feasible - should be the aim.

Children with the most mild phenotype, who did not undergo an intervention, were older at diagnosis, with less severe symptoms and lower AoV PG. In this group of less symptomatic children with relatively low gradients, waiting with intervening appeared to be a safe option in our center, and this should be explored in future studies. These VAS patients, who may experience disease (PG) stabilization, are ideally identified at the time of diagnosis, although it is currently difficult to distinguish these patients from those requiring an intervention by clinical profile.

Previous literature on the natural course of VAS in children is scattered and the majority of recommendations in current clinical practice rely on the catheterization-based 2011 AHA recommendations [11] and Second Natural History study [2]. In the former recommendations for VAS intervention are based on invasive hemodynamic assessment [11], which could limit their application in centres that primarily use echo Doppler, and, paired with current findings, underscore the need for uniform diagnosis and treatment recommendations. In the latter, a large study from 1993, VAS was progressive and progression of mild/moderate to severe VAS was especially observed in younger children [2]. Ten Harkel et al. [12] concluded that disease progression in patients with mild congenital VAS is limited and recommend regular follow-up for children without a surgical indication. If PGs are used in diagnosing VAS, it may be recommended to have a second assessment shortly after if the clinical state of the child allows it. Future research should aim for multi-center collaborations to compare outcomes following different decision-making approaches, while also evaluating outcomes of watchful waiting.

To our knowledge, the present study represents the first description of children that show regression of Doppler gradient from severe to mild/moderate VAS on serial follow-up. In pediatric pulmonary stenosis, spontaneous disease severity regression has been observed [13] and has been postulated to occur due to proper (Z-score) increase of the annulus [13]. Whether the same may apply to pediatric VAS remains to be investigated.

### 4.2 Mortality

For those with critical VAS, endocardial fibroelastosis and neonates, the risk of death before an intervention was considerably higher and long-term survival significantly lower. Little is known on the mortality risk in the trajectory between diagnosis and intervention in neonates. After AoV interventions for severe VAS, however, Brown [1] in 2003 concluded that neonates exhibited the highest in-hospital mortality of 17 %, and of up to 33 % when critical, after treatment. Contemporary literature shows a decreasing early mortality in this population [14,15], some reporting no early deaths [14]. Not performing an intervention in neonates with critical or severe VAS is thought to be associated with high mortality, but in the present study 2 out of 6 neonates without an intervention survived (33 %). The outcomes of watchful waiting in newborns with severe VAS has not been systematically evaluated to date. In the long-term, the observation in the current study that neonates have a lower survival than older children corresponds well to recent findings [[16], [17], [18]]. Survival in the 24 children whose Doppler PG regressed or stabilized without intervening was 96 % at 30 years after diagnosis, suggesting that alert, watchful observation may be a valid option in selected children diagnosed by Doppler PG.

### 4.3 Diversity of treatment pathways

The majority of children in the current study underwent at least one AoV intervention. The choice of initial and sequential treatment strategies were highly age-dependent. Notably, BV was the most commonly performed intervention, particularly as a primary choice in neonates and infants. As reintervention after BV, the Ross procedure was most common, followed by repeat BV. The Ross procedure was the second most performed intervention overall, and was only employed as a primary treatment strategy in children >1y, in line with other studies [1,3,19]. The third most commonly performed intervention was surgical AoV repair, particularly in neonates and infants. AoV repair was seldom performed after BV. To date, there is no clear consensus on whether BV or AoV repair should be the initial management of choice for children with isolated congenital AS [20,21]. Consensus opinion is that both procedures are palliative as most patients will require further interventions including AVR later in childhood or adulthood [3,22]. Although comparable early mortality (∼1–2 %), late survival and freedom from AVR are reported between BV and surgical AoV repair, AoV repair, in several studies, resulted in higher freedom from re-intervention compared to BV, particularly in neonates [20,21]. For older children, AVR might be considered as well as first intervention, as mortality risks and somatic growth are considerably lower [3,23].

In patients who underwent AVR in this study, (prosthetic) valve selection varied, with the Ross (53.7 %) and MP-AVR (32.2 %) being most commonly used. According to a recent review, in children MP-AVR is associated with lower long-term survival relative to the general age-matched population than the Ross procedure [3]. Although in theory suitable in older children, MP-AVR is infrequently considered as a primary option at our center, especially when growth is anticipated. This is well-reflected by high 15-year freedom-from-MP-AVR, ranging from 76 % in patients diagnosed at adolescent age to 100 % in patients diagnosed at neonatal age.

Other AVR options include bioprosthetic (BP-AVR) and homograft AVR (HG-AVR). These substitutes exhibit minimal thrombogenicity. However, the durability of both HG-AVR is limited due to patient outgrowth and structural deterioration [3,24]. These considerations are well-reflected in the present cohort. BP-AVR was only performed in adults as a reoperation, at a median age of 36.3 years. The median year of HG-AVR was 2003, opposed to 2011 for MP-AVR, showcasing the changing landscape of treatment decision-making. HG-AVR is now nearly abandoned. Over time (Fig. 4), BV has been increasingly used as a primary intervention, coupled with a decrease in AoV repair and the Ross procedure as primary management, which may have affected survival and reintervention outcomes in earlier decades.

Taking a lifelong perspective, staged approaches show great potential. For example, “gentle” BV in patients with diminished LV function, followed by AoV repair in the following months [15] yields good results. Additionally, a secondary Ross procedure after initial AoV repair buys children time to grow and yields satisfactory durability results and excellent survival [19]. The preferred technique should be tailored to the individual, and interventional planning at any point should consider age as a factor.

### 4.4 Limitations

Firstly, this study relied on retrospectively collected data, which may lead to missing or incomplete information.

Secondly, the findings may not be fully generalizable to other healthcare settings or populations, as they reflect the characteristics, referral patterns and treatment practices of a single center.

Thirdly, the inclusion of patients was based on Doppler PG, which may be suboptimal in some settings and could hypothetically misclassify patients with moderate VAS. However, we reviewed all diagnostic echoes of patients without an intervention, and required two successive measurements ≥4.0 m/s (64 mmHg PG) for diagnosis.

Additionally, the first option in most children represents BV at our center. Lastly, this study focused on VAS with and without additional anomalies. It is worth noting that VAS in children is part of a spectrum of obstructive conditions and can be accompanied by lesions such as aortic coarctation, the inclusion of which may negatively affect time-related outcomes in this study given a worse phenotype [25].

Lastly, this study did not provide enough granular data on ventricular physiology, e.g. hypertrophy and strain, late after watchful waiting to recommend following this approach.

## 5 Conclusions

This study highlights the vast heterogeneity of treatment pathways followed by children diagnosed with severe VAS at different ages; BV was the most common intervention in children ≤1y at diagnosis, whereas this was valve replacement in children >1y at diagnosis. Neonates show high early risk of mortality, earlier intervention and earlier reintervention compared to all other age groups. In a small proportion of children, severe VAS stabilized or regressed to mild-to-moderate AS according to serial Doppler gradients without an intervention and without symptoms, with excellent long-term survival, suggesting a potential decrease in disease severity without intervention, that should be further explored.

## CRediT authorship contribution statement

**Maximiliaan L. Notenboom:** Conceptualization, Data curation, Formal analysis, Investigation, Methodology, Project administration, Software, Validation, Visualization, Writing – original draft, Writing – review & editing. **Sencer Albayrak:** Data curation, Formal analysis, Investigation, Methodology, Project administration, Writing – original draft. **Kevin M. Veen:** Formal analysis, Investigation, Methodology, Software, Supervision, Writing – review & editing. **Ingrid van Beynum:** Formal analysis, Investigation, Resources, Supervision, Writing – review & editing. **Rebecca Swens:** Data curation, Formal analysis, Writing – review & editing. **Jolien W. Roos-Hesselink:** Investigation, Resources, Supervision, Validation, Writing – review & editing. **Pieter C. van de Woestijne:** Investigation, Resources, Supervision, Validation, Visualization, Writing – review & editing. **Jonathan R.G. Etnel:** Conceptualization, Data curation, Formal analysis, Investigation, Methodology, Project administration, Software, Supervision, Validation, Writing – review & editing. **Willem A. Helbing:** Resources, Supervision, Validation, Visualization, Writing – review & editing. **Johanna J.M. Takkenberg:** Conceptualization, Formal analysis, Investigation, Methodology, Project administration, Resources, Supervision, Validation, Writing – review & editing. **Ad J.J.C. Bogers:** Conceptualization, Formal analysis, Funding acquisition, Investigation, Methodology, Project administration, Resources, Supervision, Writing – review & editing.

## Data availability statement

All data and analytical methods will be made available upon reasonable request.

## Funding

This research was funded by the 10.13039/501100003061Erasmus University Medical Center. None of the authors received funding for this study.

## Declaration of competing interest

The authors declare that they have no known competing financial interests or personal relationships that could have appeared to influence the work reported in this paper

## References

[1] Brown J.W., Ruzmetov M., Vijay P., Rodefeld M.D., Turrentine M.W. (2003). Surgery for aortic stenosis in children: a 40-year experience. Ann Thorac Surg.

[2] Keane J.F., Driscoll D.J., Gersony W.M. (1993). Second natural history study of congenital heart defects. Results of treatment of patients with aortic valvar stenosis. Circulation.

[3] Notenboom M.L., Schuermans A., Etnel J.R.G. (2023). Paediatric aortic valve replacement: a meta-analysis and microsimulation study. Eur Heart J.

[4] Etnel J.R.G., Elmont L.C., Ertekin E. (2016). Outcome after aortic valve replacement in children: a systematic review and meta-analysis. J Thorac Cardiovasc Surg.

[5] Harris P., Kuppurao L. (2015). Quantitative doppler echocardiography. BJA Educat.

[6] Otto C.M., Nishimura R.A., Bonow R.O. (2021). 2020 ACC/AHA guideline for the management of patients with valvular heart disease: executive summary: a report of the American college of cardiology/American heart association joint committee on clinical practice guidelines. Circulation.

[7] von Elm E., Altman D.G., Egger M. (2008). The strengthening the reporting of observational studies in epidemiology (STROBE) statement: guidelines for reporting observational studies. J Clin Epidemiol.

[8] Akins C.W., Miller D.C., Turina M.I. (2008). Guidelines for reporting mortality and morbidity after cardiac valve interventions. J Thorac Cardiovasc Surg.

[9] Ross R.D. (2012). The ross classification for heart failure in children after 25 years: a review and an age-stratified revision. Pediatr Cardiol.

[10] Wang X., Notenboom M.L., Veen K.M. (2025).

[11] Feltes T.F., Bacha E., Beekman R.H. (2011). Indications for cardiac catheterization and intervention in pediatric cardiac disease: a scientific statement from the American heart association. Circulation.

[12] Harkel A.D.J.T., Berkhout M., Hop W.C., Witsenburg M., Helbing W.A. (2009). Congenital valvular aortic stenosis: limited progression during childhood. Arch Dis Child.

[13] Arain N.I., Moller J.H., Pyles L.A., Sivanandam S. (2012). "Vanishing" pulmonary valve stenosis. Ann Pediatr Cardiol.

[14] Kjellberg Olofsson C., Hanseus K., Johansson Ramgren J., Johansson Synnergren M., Sunnegårdh J. (2020). A national study of the outcome after treatment of critical aortic stenosis in the neonate. Cardiol Young.

[15] Vergnat M., Asfour B., Arenz C. (2019). Aortic stenosis of the neonate: a single-center experience. J Thorac Cardiovasc Surg.

[16] Zhu M.Z.L., Konstantinov I.E., Wu D.M., Wallace F.R.O., Brizard C.P., Buratto E. (2023;166(5):1279-1288.e1). Aortic valve repair versus the ross procedure in children. J Thorac Cardiovasc Surg.

[17] Notenboom M.L., Rhellab R., Etnel J.R.G. (2023). Aortic valve repair in neonates, infants and children: a systematic review, meta-analysis and microsimulation study. Eur J Cardio Thorac Surg.

[18] Hraška V. (2016). Neonatal aortic stenosis is a surgical disease. Semin Thorac Cardiovasc Surg Pediatr Card Surg Annu.

[19] Buratto E., Wallace F.R.O., Fricke T.A. (2020). Ross procedures in children with previous aortic valve surgery. J Am Coll Cardiol.

[20] Siddiqui J., Brizard C.P., Galati J.C. (2013). Surgical valvotomy and repair for neonatal and infant congenital aortic stenosis achieves better results than interventional catheterization. J Am Coll Cardiol.

[21] d'Udekem Y., Siddiqui J., Seaman C.S. (2013). Long-term results of a strategy of aortic valve repair in the pediatric population. J Thorac Cardiovasc Surg.

[22] Herrmann J.L., Clark A.J., Colgate C. (2020). Surgical valvuloplasty versus balloon dilation for congenital aortic stenosis in pediatric patients. World J Pediatr Congenit Heart Surg.

[23] Konstantinov I.E., Bacha E., Barron D. (2024). Optimal timing of ross operation in children: a moving target?. J Thorac Cardiovasc Surg.

[24] Takkenberg J.J.M., Klieverik L.M.A., Bekkers J.A. (2007). Allografts for aortic valve or root replacement: insights from an 18-year single-center prospective follow-up study. Eur J Cardio Thorac Surg.

[25] McCrindle B.W. (1996). Independent predictors of immediate results of percutaneous balloon aortic valvotomy in children. Valvuloplasty and angioplasty of congenital anomalies (VACA) registry investigators. Am J Cardiol.

