# Supplementary material for: Clinical course of severe congenital aortic valve stenosis in children
Source: Int J Cardiol Congenit Heart Dis. 2025 Oct 9;22:100626. doi: 10.1016/j.ijcchd.2025.100626 (PMC12593686; doi:10.1016/j.ijcchd.2025.100626)
Supplement: Multimedia component 1 [file mmc1.docx]

Clinical course of severe congenital aortic valve stenosis in children

**TEXT AND TABLES**

**Supplement 1.** STROBE checklist.

**Supplement 2.** Explanation of the modified, age-specific Ross classification.

**Supplement 3.** Elaboration of multiple imputation by chained equations and recurrent event analysis.

**Supplement 4.** Missingness per variable as a percentage at baseline.

**Supplement 5.** Correlation plot of all variables for imputation.

**Supplement 6.** Elaboration on the statistical methods for Cox proportional hazards model selection and linear mixed-effects models for echo data.

**Supplement 7.** Flowchart depicting the search process to identify patients diagnosed with severe aortic stenosis between January 1985 and February 2022. Abbreviations: HLHS, Hypoplastic left heart syndrome.

**Supplement 8.** Baseline characteristics for neonatal vs non-neonatal diagnosis.

**Supplement 9.** Detailed characterization of the 17 patients with serial Doppler gradient stabilization and 7 patients with serial Doppler gradient regression without an intervention.

**Supplement 10.** Detailed description of the causes of death before a planned aortic valve intervention in 3 neonates and 1 infant and causes for a failed intervention.

**Supplement 11.** Survival after diagnosis of severe VAS for neonates and infants (<1y) with vs without endocardial fibroelastosis, for neonates and infants (<1y) with critical vs non-critical VAS, and neonates vs non-neonates.

**Supplement 12.** Intervention details for first VAS intervention.

**Supplement 13.** Intervention details for second VAS interventions (patients with one prior intervention).

**Supplement 14.** Intervention details for third VAS interventions (patients with two prior interventions).

**Supplement 15.** Sankey plot depicting treatment pathways for all children with at least one intervention after diagnosis of severe aortic valve stenosis.

**Supplement 16.** Kaplan-Meier curve for freedom from first balloon valvuloplasty, for neonates, infants, children and teenagers.

**Supplement 17.** Kaplan-Meier curve for freedom from first surgical aortic valve repair, for neonates, infants, children and teenagers.

**Supplement 18.** Age-specific recurrent event plot for patients receiving an intervention.

**Supplement 19.** Recurrent event plot for patients receiving an intervention.

**Supplement 20.** Mean cumulative function plot for interventions across age groups.

**Supplement 21.** Evolution of fractional shortening and AoV max jet velocity.

**Online** **References.** References used in Supplementary Material.

**Supplement 1.**

**STROBE Statement**—checklist of items that should be included in reports of observational studies

|  | Item No. | Recommendation | Page  No. | Relevant text from manuscript |
| --- | --- | --- | --- | --- |
| **Title and abstract** | 1 | (*a*) Indicate the study’s design with a commonly used term in the title or the abstract | 1 | “Clinical course” |
|  |  | (*b*) Provide in the abstract an informative and balanced summary of what was done and what was found | 2 | - |
| Introduction | | | |  |
|  | 2 | Explain the scientific background and rationale for the investigation being reported | 5 | - |
| Objectives | 3 | State specific objectives, including any prespecified hypotheses | 5 | Line 104-105 |
| Methods | | | |  |
| Study design | 4 | Present key elements of study design early in the paper | 6 | - |
| Setting | 5 | Describe the setting, locations, and relevant dates, including periods of recruitment, exposure, follow-up, and data collection | 6 | Data and definitions |
| Participants | 6 | (*a*) *Cohort study*—Give the eligibility criteria, and the sources and methods of selection of participants. Describe methods of follow-up  *Case-control study*—Give the eligibility criteria, and the sources and methods of case ascertainment and control selection. Give the rationale for the choice of cases and controls  *Cross-sectional study*—Give the eligibility criteria, and the sources and methods of selection of participants | 6 | Patients  Data and definitions |
|  |  | (*b*) *Cohort study*—For matched studies, give matching criteria and number of exposed and unexposed  *Case-control study*—For matched studies, give matching criteria and the number of controls per case | N.A. | N.A. |
| Variables | 7 | Clearly define all outcomes, exposures, predictors, potential confounders, and effect modifiers. Give diagnostic criteria, if applicable | 6-7 | Data and definitions  Supplement 2 (NYHA kids) |
| Data sources/ measurement | 8* | For each variable of interest, give sources of data and details of methods of assessment (measurement). Describe comparability of assessment methods if there is more than one group | 6-7 | Data and definitions |
| Bias | 9 | Describe any efforts to address potential sources of bias | - | - |
| Study size | 10 | Explain how the study size was arrived at | 6 | Patients |

*Continued on next page*

| Quantitative variables | 11 | Explain how quantitative variables were handled in the analyses. If applicable, describe which groupings were chosen and why | 7-8 | Statistical analyses |
| --- | --- | --- | --- | --- |
| Statistical methods | 12 | (*a*) Describe all statistical methods, including those used to control for confounding | 7-8 | Statistical analyses |
|  |  | (*b*) Describe any methods used to examine subgroups and interactions | 7-8 | Statistical analyses |
|  |  | (*c*) Explain how missing data were addressed | 8 | Statistical analyses |
|  |  | (*d*) *Cohort study*—If applicable, explain how loss to follow-up was addressed  *Case-control study*—If applicable, explain how matching of cases and controls was addressed  *Cross-sectional study*—If applicable, describe analytical methods taking account of sampling strategy | 7 | Data and definitions  (modified Clark’s C) |
|  |  | (*e*) Describe any sensitivity analyses | - | - |
| Results | | | | |
| Participants | 13* | (a) Report numbers of individuals at each stage of study—eg numbers potentially eligible, examined for eligibility, confirmed eligible, included in the study, completing follow-up, and analysed | 10-13 | All Kaplan-Meier (incidence) figures, completeness of follow-up |
|  |  | (b) Give reasons for non-participation at each stage | 10-13 | Text describing deaths, lost to FUP |
|  |  | (c) Consider use of a flow diagram | Sankey plots  Supplement | Figure 4  Supplemental figures 7 and 15 |
| Descriptive data | 14* | (a) Give characteristics of study participants (eg demographic, clinical, social) and information on exposures and potential confounders | 10  Supplement | Tables 1 and 2  Supplemental tables 8 and 9 |
|  |  | (b) Indicate number of participants with missing data for each variable of interest | 10 | Tables 1 and 2, Supplement |
|  |  | (c) *Cohort study*—Summarise follow-up time (eg, average and total amount) | 10 | Text with FUP |
| Outcome data | 15* | *Cohort study*—Report numbers of outcome events or summary measures over time | 10-13 | See text, Mortality, Interventions |
|  |  | *Case-control study—*Report numbers in each exposure category, or summary measures of exposure | N.A. | N.A. |
|  |  | *Cross-sectional study—*Report numbers of outcome events or summary measures | N.A. | N.A. |
| Main results | 16 | (*a*) Give unadjusted estimates and, if applicable, confounder-adjusted estimates and their precision (eg, 95% confidence interval). Make clear which confounders were adjusted for and why they were included |  | See all results. |
|  |  | (*b*) Report category boundaries when continuous variables were categorized | - | See all results. |
|  |  | (*c*) If relevant, consider translating estimates of relative risk into absolute risk for a meaningful time period | - | - |

*Continued on next page*

| Other analyses | 17 | Report other analyses done—eg analyses of subgroups and interactions, and sensitivity analyses | 10-11 | “Patients” and “Survival” |
| --- | --- | --- | --- | --- |
| Discussion | | | | |
| Key results | 18 | Summarise key results with reference to study objectives | 14 | “This single-center … AoV intervention” |
| Limitations | 19 | Discuss limitations of the study, taking into account sources of potential bias or imprecision. Discuss both direction and magnitude of any potential bias | 17-18 | Discussion section, Limitations |
| Interpretation | 20 | Give a cautious overall interpretation of results considering objectives, limitations, multiplicity of analyses, results from similar studies, and other relevant evidence | 14-15  16-17 | Discussion section: Natural course and Diversity of treatment pathways |
| Generalisability | 21 | Discuss the generalisability (external validity) of the study results | 15 | Discussion section: Natural course |
| Other information | |  | | |
| Funding | 22 | Give the source of funding and the role of the funders for the present study and, if applicable, for the original study on which the present article is based | 1 | Title page |

*Give information separately for cases and controls in case-control studies and, if applicable, for exposed and unexposed groups in cohort and cross-sectional studies.

**Note:** An Explanation and Elaboration article discusses each checklist item and gives methodological background and published examples of transparent reporting. The STROBE checklist is best used in conjunction with this article (freely available on the Web sites of PLoS Medicine at http://www.plosmedicine.org/, Annals of Internal Medicine at http://www.annals.org/, and Epidemiology at http://www.epidem.com/). Information on the STROBE Initiative is available at www.strobe-statement.org.

**Supplement 2.**

Ross classification for heart failure in children

To categorically assess the heart failure severity in all children, the modified age-specific Ross Heart Failure Classification was used(1). Each electronic patient file was comprehensively screened and the patient’s heart failure severity at diagnosis and during all repeated follow-up visits was graded based on the medical record. The modified, age-specific Ross Classification grades adhered to were: 1) All ages: no limitations or symptoms; 2) Infants: Mild tachypnea or diaphoresis with feeding, Older children: mild to moderate dyspnea on exertion; 3) Infants: growth failure and marked tachypnea or diaphoresis with feeding, Older children: marked dyspnea on exertion; 4) All ages: symptoms at rest such as tachypnea, retractions, grunting, or diaphoresis.

**Supplement 3.**

Multiple Imputation by Chained Equations

Selected baseline variables with <30% missing values were imputed; >30% missing values was considered excessive missingness. Out of 69 variables, 18 (26.1%) had <30% missingness. An exception was made for the variable Fractional Shortening (50.8% missing), because it is clinically relevant in the context of severe AS. Imputations were carried out based on other observed baseline variables. In the case of highly correlated variables, the variable with the highest clinical relevance was chosen as the predictor to avoid multicollinearity (Online Material, Table S2). Correlations were tested with Pearson R or Spearman Rho, as appropriate. Using this method five imputed datasets were created, using 25 iterations each. The imputations were subsequently visually inspected by strip plots and density plots. No systematic deviations were noted between the imputed data and complete data. Analyses were done on each dataset separately and finally pooled according to Rubin’s rules(2).

**Supplement 4.**

| Baseline variable | Missing count | Missing percentage |
| --- | --- | --- |
| Date of birth | 0 | 0.00 |
| Sex* | 0 | 0.00 |
| Birth weight | 98 | 37.12 |
| Gestational age at birth | 112 | 42.42 |
| Date of diagnosis | 0 | 0.00 |
| Age at diagnosis* | 0 | 0.00 |
| Weight* | 21 | 7.95 |
| Height* | 47 | 17.80 |
| Body surface Area* | 48 | 18.18 |
| AV cusp morphology* | 43 | 16.29 |
| Etiology | 7 | 2.65 |
| Associated cardiac lesions | 29 | 10.98 |
| Concomitant SAS* | 29 | 10.98 |
| Ross classification* | 18 | 6.82 |
| NYHA classification | 131 | 49.62 |
| Systolic blood pressure | 92 | 34.85 |
| Diastolic blood pressure | 105 | 39.77 |
| Heart rate* | 59 | 22.35 |
| Heart rhythm | 38 | 14.39 |
| Respiratory rate | 169 | 64.02 |
| Ventilator use* | 12 | 4.55 |
| Diuretic use* | 13 | 4.92 |
| Inotropic use* | 13 | 4.92 |
| AV Vmax jet velocity* | 16 | 6.06 |
| AV peak gradient | 16 | 6.06 |
| Fractional shortening* | 134 | 50.76 |
| Left ventricular ejection fraction | 260 | 98.48 |
| Aortic regurgitation grade | 188 | 71.21 |
| Aortic regurgitation pressure-halftime | 257 | 97.35 |
| Aortic regurgitation width | 264 | 100.00 |
| Aortic regurgitation vena contracta | 264 | 100.00 |
| IVSd | 143 | 54.17 |
| IVSs | 174 | 65.91 |
| LVIDd | 140 | 53.03 |
| LVIDs | 143 | 54.17 |
| LVPWd | 142 | 53.79 |
| LVPWs | 175 | 66.29 |
| LV Mass | 218 | 82.58 |
| Aortic root diameter | 216 | 81.82 |
| LA diameter | 233 | 88.26 |
| Ratio LA/Aortic root diameter | 245 | 92.80 |
| MV E-wave velocity | 210 | 79.55 |
| MV A-wave velocity | 214 | 81.06 |
| MV E/A wave ratio | 226 | 85.61 |
| MV deceleration time | 255 | 96.59 |
| TV E-wave velocity | 231 | 87.50 |
| TV A-wave velocity | 235 | 89.02 |
| TV E/A wave ratio | 236 | 89.39 |
| PA Vmax velocity | 216 | 81.82 |
| PA peak gradient | 221 | 83.71 |

*SAS: Subvalvular AS, CoA: Aortic coarctation; VSD: Ventricular septum defect; ASD: Atrial septum defect; PDA: Patent ductus arteriosus; MV: Mitral valve; PV: Pulmonary valve; AV: Aortic valve; IVSd: Interventricular septal end diastole; IVSs: Interventricular septal end systole; LVIDd: Left ventricular internal diameter end diastole; LVIDs: Left ventricular internal diameter end systole; LVPWd: Left ventricular posterior wall end diastole; LVPWs: Left ventricular posterior wall end systole; LV: Left ventricle; TV: Tricuspid valve; PA: Pulmonary artery. Variables denoted with “*” were used for multiple imputation by chained equations.*

**Supplement 5.**

|  | **id** | **time** | **event** | **BSA** | **height** | **weight** | **AV cusps** | **hr** | **fs** | **AS jet** | **AR class** | **SAS** | **ross class** | **AS peak** | **inotropic** | **ventilator** | **diuretic** | **sex** | **diagnosis year** | **age** | **death** |
| --- | --- | --- | --- | --- | --- | --- | --- | --- | --- | --- | --- | --- | --- | --- | --- | --- | --- | --- | --- | --- | --- |
| **id** | 1,00 | -0,21 | -0,05 | -0,15 | -0,16 | -0,20 | -0,10 | 0,21 | 0,02 | -0,12 | -0,01 | 0,20 | 0,14 | -0,12 | 0,09 | 0,15 | 0,05 | 0,07 | 0,59 | -0,24 | 0,08 |
| **time** | -0,21 | 1,00 | -0,38 | 0,55 | 0,55 | 0,58 | 0,10 | -0,51 | 0,14 | -0,17 | 0,19 | 0,03 | -0,45 | -0,17 | -0,19 | -0,19 | -0,19 | 0,08 | -0,24 | 0,57 | -0,12 |
| **event** | -0,05 | -0,38 | 1,00 | -0,15 | -0,14 | -0,20 | 0,11 | 0,11 | -0,15 | 0,15 | -0,21 | 0,00 | 0,17 | 0,16 | 0,10 | 0,09 | 0,11 | -0,07 | -0,14 | -0,15 | -0,07 |
| **BSA** | -0,15 | 0,55 | -0,15 | 1,00 | 0,99 | 1,00 | 0,22 | -0,86 | 0,29 | -0,06 | 0,40 | -0,02 | -0,33 | -0,07 | -0,27 | -0,21 | -0,22 | 0,14 | -0,41 | 0,98 | -0,22 |
| **height** | -0,16 | 0,55 | -0,14 | 0,99 | 1,00 | 0,98 | 0,22 | -0,87 | 0,27 | -0,05 | 0,40 | -0,04 | -0,33 | -0,06 | -0,28 | -0,22 | -0,23 | 0,14 | -0,42 | 0,98 | -0,21 |
| **weight** | -0,20 | 0,58 | -0,20 | 1,00 | 0,98 | 1,00 | 0,21 | -0,82 | 0,35 | 0,00 | 0,42 | -0,03 | -0,43 | -0,01 | -0,22 | -0,24 | -0,22 | 0,12 | -0,43 | 0,97 | -0,24 |
| **AV cusps** | -0,10 | 0,10 | 0,11 | 0,22 | 0,22 | 0,21 | 1,00 | -0,15 | 0,15 | 0,10 | 0,23 | 0,08 | -0,04 | 0,10 | -0,17 | -0,11 | 0,03 | -0,03 | -0,19 | 0,17 | -0,15 |
| **hr** | 0,21 | -0,51 | 0,11 | -0,86 | -0,87 | -0,82 | -0,15 | 1,00 | -0,28 | 0,00 | -0,37 | -0,02 | 0,35 | 0,01 | 0,23 | 0,11 | 0,23 | -0,12 | 0,48 | -0,86 | 0,14 |
| **fs** | 0,02 | 0,14 | -0,15 | 0,29 | 0,27 | 0,35 | 0,15 | -0,28 | 1,00 | 0,17 | 0,11 | 0,16 | -0,25 | 0,15 | -0,13 | -0,21 | -0,12 | -0,10 | -0,07 | 0,33 | -0,09 |
| **AS jet** | -0,12 | -0,17 | 0,15 | -0,06 | -0,05 | 0,00 | 0,10 | 0,00 | 0,17 | 1,00 | -0,13 | 0,19 | -0,02 | 0,99 | -0,12 | -0,23 | -0,08 | 0,04 | -0,21 | 0,00 | -0,01 |
| **AR class** | -0,01 | 0,19 | -0,21 | 0,40 | 0,40 | 0,42 | 0,23 | -0,37 | 0,11 | -0,13 | 1,00 | 0,05 | -0,20 | -0,15 | -0,07 | -0,06 | -0,09 | -0,05 | -0,01 | 0,40 | -0,07 |
| **SAS** | 0,20 | 0,03 | 0,00 | -0,02 | -0,04 | -0,03 | 0,08 | -0,02 | 0,16 | 0,19 | 0,05 | 1,00 | 0,00 | 0,19 | -0,05 | -0,05 | -0,06 | -0,01 | 0,10 | -0,04 | 0,12 |
| **Ross class** | 0,14 | -0,45 | 0,17 | -0,33 | -0,33 | -0,43 | -0,04 | 0,35 | -0,25 | -0,02 | -0,20 | 0,00 | 1,00 | -0,01 | 0,17 | 0,31 | 0,30 | -0,09 | 0,11 | -0,38 | 0,29 |
| **AS peak** | -0,12 | -0,17 | 0,16 | -0,07 | -0,06 | -0,01 | 0,10 | 0,01 | 0,15 | 0,99 | -0,15 | 0,19 | -0,01 | 1,00 | -0,12 | -0,23 | -0,08 | 0,04 | -0,21 | -0,01 | -0,01 |
| **inotropic** | 0,09 | -0,19 | 0,10 | -0,27 | -0,28 | -0,22 | -0,17 | 0,23 | -0,13 | -0,12 | -0,07 | -0,05 | 0,17 | -0,12 | 1,00 | 0,36 | 0,46 | -0,08 | 0,13 | -0,24 | 0,08 |
| **ventilator** | 0,15 | -0,19 | 0,09 | -0,21 | -0,22 | -0,24 | -0,11 | 0,11 | -0,21 | -0,23 | -0,06 | -0,05 | 0,31 | -0,23 | 0,36 | 1,00 | 0,30 | -0,01 | 0,13 | -0,25 | 0,18 |
| **diuretic** | 0,05 | -0,19 | 0,11 | -0,22 | -0,23 | -0,22 | 0,03 | 0,23 | -0,12 | -0,08 | -0,09 | -0,06 | 0,30 | -0,08 | 0,46 | 0,30 | 1,00 | -0,07 | 0,10 | -0,21 | 0,05 |
| **sex** | 0,07 | 0,08 | -0,07 | 0,14 | 0,14 | 0,12 | -0,03 | -0,12 | -0,10 | 0,04 | -0,05 | -0,01 | -0,09 | 0,04 | -0,08 | -0,01 | -0,07 | 1,00 | 0,02 | 0,08 | -0,07 |
| **diagnosis year** | 0,59 | -0,24 | -0,14 | -0,41 | -0,42 | -0,43 | -0,19 | 0,48 | -0,07 | -0,21 | -0,01 | 0,10 | 0,11 | -0,21 | 0,13 | 0,13 | 0,10 | 0,02 | 1,00 | -0,50 | 0,03 |
| **age** | -0,24 | 0,57 | -0,15 | 0,98 | 0,98 | 0,97 | 0,17 | -0,86 | 0,33 | 0,00 | 0,40 | -0,04 | -0,38 | -0,01 | -0,24 | -0,25 | -0,21 | 0,08 | -0,50 | 1,00 | -0,21 |
| **death** | 0,08 | -0,12 | -0,07 | -0,22 | -0,21 | -0,24 | -0,15 | 0,14 | -0,09 | -0,01 | -0,07 | 0,12 | 0,29 | -0,01 | 0,08 | 0,18 | 0,05 | -0,07 | 0,03 | -0,21 | 1,00 |

*BSA: Body surface area; AV: Aortic valve; Hr; heart rate; Fs: Fractional shortening; AS jet: AV Vmax; AR: aortic regurgitation; SAS: Subvalvular AS; AS peak: AV peak gradient. Yellow cells: correlations <-0.7 and red cells: correlations >0.7 considered as high correlation. The correlation plot shows strong correlations among variables age, AV Vmax, AV peak gradient, weight, height, BSA, and heart rate. As a result, to prioritize highly relevant variables such as age and AV Vmax, variables height, weight, BSA, heart rate, and AV peak gradient were excluded from the multivariable Cox model.*

**Supplement 6.**

Cox model selection

No multivariable model was selected for mortality to avoid overfitting, as there was a lack of events (deaths). In multivariable modelling, all variables with a p-value <0.05 in the univariable mode l were included in the multivariable model (en-bloc forward), excluding variables showcasing strong correlation with other variables. Correlation between variables of ≥0.70 or ≤-0.70, according to Spearman’s/Pearson’s correlation coefficient, was defined as strong correlation, and only one of the two variables was retained in the multivariable model. In case of significant correlation, defined as listed above, the most clinically relevant parameter determined by the research team was prioritized. Additionally, a minimum of ten events per risk factor was used as a rule of thumb to avoid overfitting in multivariable Cox regression. Analyses were done on each imputed dataset separately and pooled according to Rubin’s rule. The final variables included in the multivariable model for time to first intervention were age group, valve morphology, VAS jet velocity, Ross class 3/4, ventilator/inotropics/diuretics use, fractional shortening, year of diagnosis and presence of endocardial fibroelastosis.

Mixed-effects models

To model valve and ventricular function over time, age-specific linear mixed-effects models were constructed and applied to the longitudinal echocardiographic data. Random intercepts and random slopes were included at the level of the patient. Nonlinear terms were considered in the fixed-effects part of the models. Subgroup analyses were performed for patients without an intervention versus patients with an intervention, and for age groups at time of diagnosis, by including interaction terms with time.

**Supplement 7.**


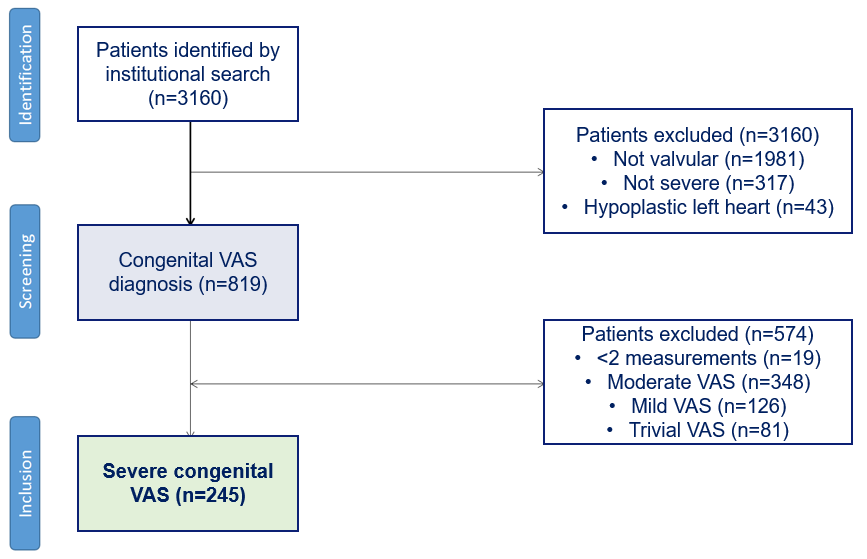


**Supplement 8.**

|  | **All patients** (100%)  n=245 | **Neonatal** (21.6%)  n=53 | **Non-neonatal** (30.3%)  n=192 | **P-Value** |  |
| --- | --- | --- | --- | --- | --- |
| Age, y | 0.94 [0.10, 6.23] | 0.01 [0.00, 0.05] | 3.03 [0.28, 7.92] | **<0.001** |  |
| Male | 177 (72.2) | 33 (62.3) | 144 (75.0) | 0.097 |  |
| Weight, kg | 8.50 [4.20, 21.80] | 3.38 [2.94, 3.85] | 13.30 [5.84, 25.30] | **<0.001** |  |
| Height, cm | 80.50 [56.58, 120.62] | 50.50 [48.50, 52.00] | 99.00 [62.50, 128.20] | **<0.001** |  |
| BSA, m2 | 0.48 [0.27, 0.88] | 0.21 [0.19, 0.23] | 0.64 [0.32, 0.99] | **<0.001** |  |
| Aortic valve morphology^ |  |  |  | **0.001** |  |
| Unicuspid | 10 (4.9) | 7 (17.1) | 3 (1.8) | **<0.001** |  |
| Bicuspid | 152 (74.5) | 28 (68.3) | 124 (76.1) | 0.411 |  |
| Tricuspid | 42 (20.6) | 6 (14.6) | 36 (22.1) | 0.402 |  |
| Ross functional classification |  |  |  | **<0.001** |  |
| Ross class I | 152 (68.2) | 15 (34.1) | 137 (76.5) | **0.007** |  |
| Ross class II | 28 (12.6) | 6 (13.6) | 22 (12.3) | 0.489 |  |
| Ross class III | 13 (5.8) | 2 (4.5) | 11 (6.1) | 0.356 |  |
| Ross class IV | 30 (13.5) | 21 (47.7) | 9 (5.0) | **0.013** |  |
| Subaortic stenosis | 19 (7.8) | 2 (3.8) | 17 (8.9) | 0.382 |  |
| Critical VAS | 18 (7.3) | 13 (24.5) | 5 (2.6) | **<0.001** |  |
| Concomitant anomalies | 73 (29.8) | 31 (58.5) | 42 (21.9) | **<0.001** |  |
| EFE | 18 (7.3) | 17 (32.1) | 1 (0.5) | **<0.001** |  |
| CoA | 29 (11.8) | 6 (11.3) | 23 (12.0) | 1.000 |  |
| VSD | 13 (5.3) | 2 (3.8) | 11 (5.7) | 0.740 |  |
| MV dysfunction (grade ≥3) | 25 (10.2) | 11 (20.8) | 14 (7.3) | **0.008** |  |
| PV dysfunction (grade ≥3) | 4 (1.6) | 1 (1.9) | 3 (1.6) | 1.000 |  |
| Ventilator use | 7 (3.0) | 7 (13.7) | 0 (0.0) | **<0.001** |  |
| Diuretics use | 12 (5.2) | 5 (9.8) | 7 (3.9) | 0.183 |  |
| Inotropics use | 9 (3.9) | 6 (11.8) | 3 (1.7) | **0.004** |  |
| Aortic valve Vmax, m/s | 4.34 [4.03, 4.81] | 4.19 [3.40, 4.65] | 4.40 [4.10, 4.89] | **0.004** |  |
| Vmax below 4.0 m/s | 20 (8.7) | 17 (39.5) | 3 (1.6) | **<0.001** |  |
| Vmax above 5.0 m/s | 53 (23.1) | 8 (18.6) | 45 (24.2) | 0.560 |  |

**Supplement 9.**

| **Patient** | **Age**, y | **Sex** | **Group** | **Vmax #1** | **Concomitant anomalies** | **Interventions** (all <diagnosis) | **Clinical presentation** | **Current age,** y | **FS**, % | **LVEDD** | **LVESD** |  |  |
| --- | --- | --- | --- | --- | --- | --- | --- | --- | --- | --- | --- | --- | --- |
| 1 | 6.6 | M | Stable | 440.0 | Aortic coarctation, mild mitral stenosis | Coarctectomy (end-to-end) | No symptoms, no LVH, normal EF, normal ECG | 8.1 | 50.4 | 4.0 | 2.0 |  |  |
| 2^ | 0.1 | M | Stable | 400.0 | PFO (L-R shunt), Narrow arch | None | No symptoms, no LVH, normal EF, normal ECG | 5.2 | 34.4 | 3.3 | 2.1 |  |  |
| 3 | 2.1 | F | Stable | 411.2 | Aortic arch hypoplasia | Aortic arch augmentation | No symptoms, no LVH, normal EF, normal ECG | 3.2 | - | - | - |  |  |
| 4 | 0.2 | M | Stable | 403.1 | Small muscular VSD | None | No symptoms, no LVH, normal EF, normal ECG | 1.9 | 49.0 | - | - |  |  |
| 5 | 0.5 | M | Stable | 403.0 | Congenital rubella, PDA, microphtalmia | None | No symptoms, no LVH, normal EF, normal ECG | 2.0 | - | - | - |  |  |
| 6 | 15.4 | M | Stable | 433.0 | None | None | No symptoms, no LVH, normal EF, normal ECG | 17.5 | 46.7 | 5.7 | 3.1 |  |  |
| 7 | 4.2 | M | Stable | 400.0 | None | None | No symptoms, no LVH, normal EF, normal ECG | 6.3 | 37.0 | 4.1 | 2.6 |  |  |
| 8 | 4.7 | M | Stable | 400.0 | None | None | No symptoms, no LVH, normal EF, normal ECG | 31.7 | 29.0 | 4.2 | 3.0 |  |  |
| 9 | 0.3 | M | Stable | 450.0 | None | None | No symptoms, no LVH, normal EF, normal ECG | 5.7 | 39.0 | 3.9 | 2.3 |  |  |
| 10 | 13.2 | M | Stable | 422.0 | None | None | No symptoms, no LVH, normal EF, normal ECG | 16.2 | 42.0 | 5.5 | 3.2 |  |  |
| 11 | 0.5 | M | Stable | 400.0 | None | None | No symptoms, no LVH, normal EF, normal ECG | 17.0 | 31.0 | 5.1 | 3.5 |  |  |
| 13 | 4.8 | M | Stable | 448.0 | None | None | No symptoms, no LVH, normal EF, normal ECG | 8.3 | 44.0 | 3.9 | 2.2 |  |  |
| 12 | 3.8 | M | Stable | 400.0 | None | None | No symptoms, no LVH, normal EF, normal ECG | 29.7 | EF: 65% | - | 4.4 |  |  |
| 14 | 0.9 | M | Stable | 434.0 | None | None | No symptoms, no LVH, normal EF, normal ECG | 4.2 | 40.0 | 3.4 | 2.0 | |  |
| 15 | 1.9 | M | Stable | 460.0 | None | None | No symptoms, no LVH, normal EF, normal ECG | 26.5 | * | 4.8 | - | |  |
| 16 | 3.6 | M | Stable | 400.0 | None | None | No symptoms, no LVH, normal EF, normal ECG | 4.7 | 38.0 | 3.4 | 2.0 | |  |
| 17 | 3.8 | F | Stable | 402.0 | None | None | No symptoms, no LVH, normal EF, normal ECG | 31.3 | 49.0 (EF: 60%) | 5.3 | 2.7 | |  |
| 18 | 0.2 | F | Regression | 460.0 | Mini-PDA | None | No symptoms, no LVH, normal EF, normal ECG | 0.9 | 40.3 | 2.6 | 1.6 | |  |
| 19 | 13.1 | F | Regression | 420.0 | None | None | No symptoms, no LVH, normal EF, normal ECG | 26.9 | 46.0 | 4.6 | 2.5 | |  |
| 20 | 0.1 | F | Regression | 430.0 | None | None | No symptoms, no LVH, normal EF, normal ECG | 21.5 | 42.0 (EF: 58%) | 5.0 | 2.9 | |  |
| 21 | 12.9 | M | Regression | 400.0 | None | None | No symptoms, no LVH, normal EF, normal ECG | 27.0 | - | 6.2 | - | |  |
| 22 | 13.6 | M | Regression | 413.0 | None | None | No symptoms, no LVH, normal EF, normal ECG | 18.9 | - | 4.7 | - | |  |
| 23 | 0.3 | F | Regression | 480.0 | None | None | No symptoms, no LVH, normal EF, normal ECG | 16.7 | 46.0 | 3.7 | 2.0 | |  |
| 24 | 6.7 | M | Regression | 400.0 | None | None | No symptoms, no LVH, normal EF, normal ECG | 16.7 | - | 4.6 | - | |  |
| ^ Patient now has a very recent measurement of Vmax 5.1m/s, still asymptomatic. * Five years prior (age 21.5), FS was 39.0% | | | | | | | |  |  |  |  | | |

**Supplement 10.**

Death before a planned intervention

The infant (year of death: 1991) that died had progressive aortic valve stenosis with severe pulmonary stenosis and biventricular hypertrophy, who had a cardiac arrest during preoperative anesthesia induction. One neonate (year of death: 1995) had significant concomitant mitral and tricuspid regurgitation with a patent duct, who died of total cardiac output loss (forward and backward failure), unresponsive to endotracheal medication administration and adrenaline. Another neonate (year of death: 2019) had concomitant pulmonary stenosis with endocardial fibroelastosis, precluding a Ross procedure, and this neonate died suddenly after feeding. The third neonate (year of death: 2020) had severe left ventricular failure, with low-flow, low-gradient aortic valve stenosis with mitral valve stenosis and endocardial fibroelastosis. Her systemic circulation was duct-dependent.

Children with unfeasible/failed intervention

One of these children ended up in the asymptomatic severe VAS group and is still alive. The other two children died shortly after diagnosis, given no remaining therapeutic options. The reasons were as follows: 1) A BV was planned in a 2-week-old neonate (1990) but did not succeed due to technical issues, which was also complicated by a large femoral thrombus, unresponsive of heparin and thrombolytic therapy; 12 hours after cessation of thrombolytic therapy, this neonate died due to a massive bleeding from the right groin. 2) A 3-year-old child with multiple congenital defects, including hypospadias, duodenal stenosis with Meckel’s diverticulum, severe psychomotor retardation and severe dysplastic valvar and moderate sub valvar AS was evaluated. At neonatal age (1994), he had his interrupted aortic arch and VSD repaired at our institution. In 1997, BV was deemed unfeasible, and, together with the parents, it was decided not to perform open surgery as this would pose a major procedure with the possibility of further deterioration. A DNR was signed. He succumbed two years later at the age of 5 years.

**Supplement 11.**

**
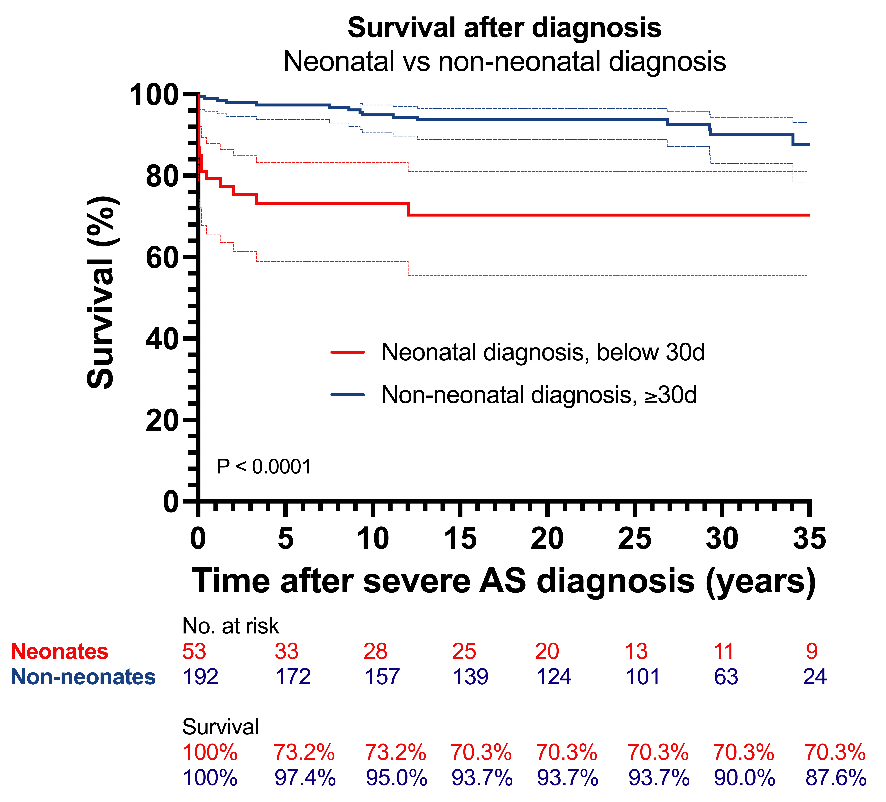
**

**
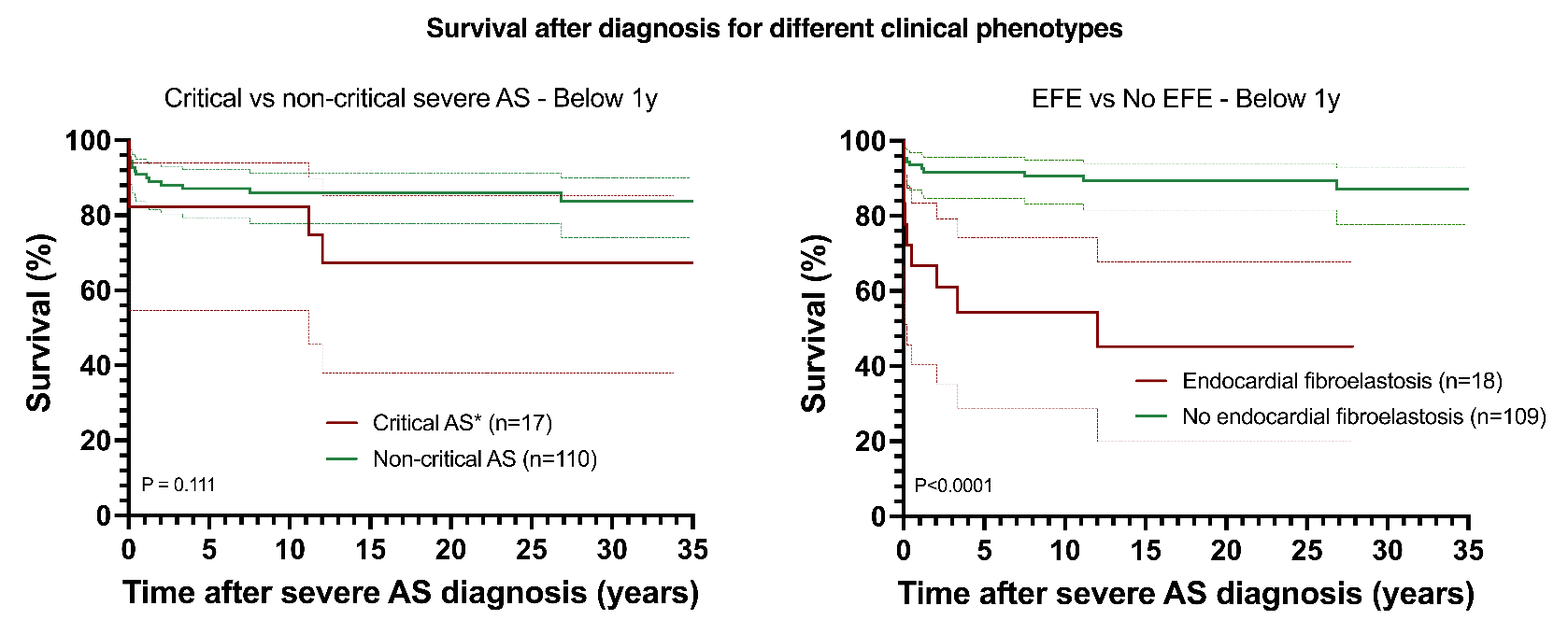

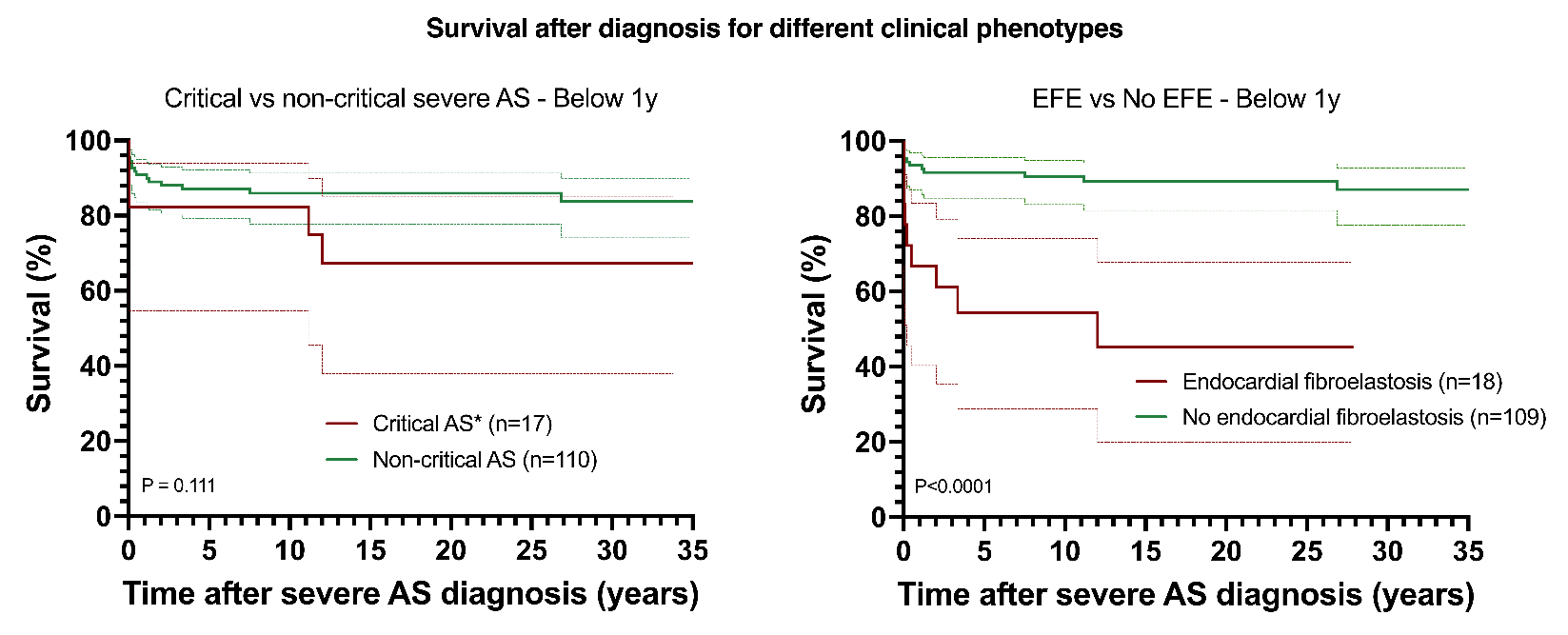
**

**Supplement 12.**

|  | **All patients** (100%) | **Neonates**  (22.3%) | **Infants**  (30.3%) | **Children**  (33.6%) | **Teenagers**  (13.7%) | **P-Value*** |
| --- | --- | --- | --- | --- | --- | --- |
| Number of interventions | 211 | 47 | 64 | 71 | 29 |  |
| Time to first intervention, y | 0.17 [0.03, 1.17] | 0.01 [0.00, 0.03] | 0.05 [0.03, 0.27] | 0.82 [0.31, 4.73] | 0.88 [0.20, 5.06] | **<0.001** |
| Procedures, n (%) |  |  |  |  |  |  |
| BV | 138 (65.4) | 37 (78.7) | 53 (82.8) | 40 (56.3) | 8 (27.6) | **<0.001** |
| SAV | 43 (20.4) | 10 (21.3) | 11 (17.2) | 18 (25.4) | 4 (13.8) | 0.516 |
| Concomitant SAS resection | 7 (3.3) | 0 (0.0) | 0 (0.0) | 7 (9.9) | 0 (0.0) | **0.003** |
| AVR | 29 (13.7) | 0 (0.0) | 0 (0.0) | 12 (16.9) | 17 (58.6) |  |
| Ross procedure | 12 (5.7) | 0 (0.0) | 0 (0.0) | 6 (8.5) | 6 (20.7) | **<0.001** |
| MP-AVR | 12 (5.7) | 0 (0.0) | 0 (0.0) | 4 (5.6) | 8 (27.6) | **<0.001** |
| HG-AVR | 5 (2.4) | 0 (0.0) | 0 (0.0) | 2 (2.8) | 3 (10.3) | **0.013** |
| (Re-)intervention etiology, n (%) |  |  |  |  |  | 0.435 |
| Congenital | 206 (98.6) | 44 (97.8) | 64 (100.0) | 70 (98.6) | 28 (96.6) |  |
| Endocarditis | 1 (0.5) | 0 (0.0) | 0 (0.0) | 1 (1.4) | 0 (0.0) |  |
| Valve-dysfunction post BV | 2 (1.0) | 1 (2.2) | 0 (0.0) | 0 (0.0) | 1 (3.4) |  |

** P-value applies for columns of age groups (neonates, infants, children, teenagers). BV: balloon aortic valvuloplasty; SAV: surgical aortic valvotomy / aortic valve repair; VSRR: valve sparing aortic root replacement; SAS: subvalvular AS; AVR: aortic valve replacement; MP-AVR: mechanical aortic valve replacement; BP-AVR: bioprosthetic aortic valve replacement; HG-AVR: homograft aortic valve replacement*

**Supplement 13.**

|  | **All patients**  (100%) | **47 Neonates**  (22.3%) | **64 Infants**  (30.3%) | **71 Children**  (33.6%) | **29 Teenagers**  (13.7%) | **P-Value*** |
| --- | --- | --- | --- | --- | --- | --- |
| Number of interventions | 122 | 36 | 30 | 40 | 16 |  |
| Time to second intervention, y | 4.7 [0.6, 10.7] | 0.3 [0.1, 5.5] | 6.1 [1.5, 11.1] | 5.2 [2.3, 11.3] | 8.4 [3.9, 13.0] | **<0.001** |
| Procedures, n (%) |  |  |  |  |  |  |
| BV | 46 (37.7) | 17 (47.2) | 10 (33.3) | 16 (40.0) | 3 (18.8) | 0.244 |
| SAV | 8 (6.6) | 1 (2.8) | 5 (16.7) | 2 (5.0) | 0 (0.0) | 0.068 |
| VSRR | 3 (2.5) | 1 (2.8) | 0 (0.0) | 1 (2.5) | 1 (6.2) | 0.630 |
| Concomitant SAS resection | 3 (2.5) | 1 (2.8) | 1 (3.3) | 1 (2.5) | 0 (0.0) | 0.916 |
| AVR | 65 (53.3) | 17 (47.2) | 16 (53.3) | 20 (50.0) | 12 (75.0) |  |
| Ross procedure | 41 (33.6) | 15 (41.7) | 8 (26.7) | 12 (30.0) | 6 (37.5) | 0.565 |
| MP-AVR | 16 (13.1) | 1 (2.8) | 5 (16.7) | 6 (15.0) | 4 (25.0) | 0.121 |
| BP-AVR | 1 (0.8) | 0 (0.0) | 0 (0.0) | 0 (0.0) | 1 (6.2) | 0.083 |
| HG-AVR | 7 (5.7) | 1 (2.8) | 3 (10.0) | 2 (5.0) | 1 (6.2) | 0.651 |
| (Re-)intervention etiology, n (%) |  |  |  |  |  | **0.015** |
| Congenital | 7 (5.8) | 5 (13.9) | 2 (6.7) | 0 (0.0) | 0 (0.0) |  |
| Endocarditis | 6 (5.0) | 0 (0.0) | 1 (3.3) | 2 (5.1) | 3 (20.0) |  |
| SVD | 38 (31.7) | 7 (19.4) | 8 (26.7) | 15 (38.5) | 8 (53.3) |  |
| NSVD | 4 (3.3) | 1 (2.8) | 2 (6.7) | 1 (2.6) | 0 (0.0) |  |
| Valve-dysfunction post BV | 65 (54.2) | 23 (63.9) | 17 (56.7) | 21 (53.8) | 4 (26.7) |  |

** P-value applies for columns of age groups (neonates, infants, children, Teenagers). BV: balloon aortic valvuloplasty; SAV: surgical aortic valvotomy / aortic valve repair; VSRR: valve sparing aortic root replacement; SAS: subvalvular AS; AVR: aortic valve replacement; MP-AVR: mechanical aortic valve replacement; BP- AVR: bioprosthetic aortic valve replacement; HG-AVR: homograft aortic valve replacement.*

**Supplement 14.**

|  | **All 211 patients**  (100%) | **47 Neonates**  (22.3%) | **64 Infants**  (30.3%) | **71 Children**  (33.6%) | **29 Teenagers**  (13.7%) | **P-Value*** |
| --- | --- | --- | --- | --- | --- | --- |
| Number of interventions | 57 | 17 | 15 | 19 | 6 |  |
| Time to ≥third intervention, y | 11.4 [1.2, 19.2] | 3.5 [0.3, 16.1] | 5.4 [0.5, 14.5] | 11.6 [4.9, 22.9] | 17.6 [14.6, 18.9] | 0.055 |
| BV | 6 (10.5) | 2 (11.8) | 2 (13.3) | 2 (10.5) | 0 (0.0) | 0.835 |
| SAV | 4 (7.0) | 2 (11.8) | 1 (6.7) | 1 (5.3) | 0 (0.0) | 0.769 |
| VSRR | 2 (3.5) | 1 (5.9) | 0 (0.0) | 1 (5.3) | 0 (0.0) | 0.748 |
| Concomitant SAS resection | 2 (3.5) | 0 (0.0) | 0 (0.0) | 2 (10.5) | 0 (0.0) | 0.246 |
| AVR | 45 (78.9) | 12 (70.6) | 12 (80.0) | 15 (78.9) | 6 (100.0) |  |
| Ross procedure | 22 (38.6) | 8 (47.1) | 8 (53.3) | 5 (26.3) | 1 (16.7) | 0.229 |
| MP-AVR | 18 (31.6) | 1 (5.9) | 4 (26.7) | 8 (42.1) | 5 (83.3) | **0.003** |
| BP-AVR | 2 (3.5) | 1 (5.9) | 0 (0.0) | 1 (5.3) | 0 (0.0) | 0.748 |
| HG-AVR | 3 (5.3) | 2 (11.8) | 0 (0.0) | 1 (5.3) | 0 (0.0) | 0.456 |
| (Re-)intervention etiology, n (%) |  |  |  |  |  | 0.425 |
| Congenital | 1 (1.8) | 1 (5.9) | 0 (0.0) | 0 (0.0) | 0 (0.0) |  |
| Endocarditis | 1 (1.8) | 0 (0.0) | 1 (6.7) | 0 (0.0) | 0 (0.0) |  |
| SVD | 24 (42.1) | 6 (35.3) | 4 (26.7) | 9 (47.4) | 5 (83.3) |  |
| NSVD | 6 (10.5) | 2 (11.8) | 1 (6.7) | 3 (15.8) | 0 (0.0) |  |
| Valve-dysfunction post BV | 25 (43.9) | 8 (47.1) | 9 (60.0) | 7 (36.8) | 1 (16.7) |  |

** P-value applies for columns of age groups (neonates, infants, children, Teenagers). BV: balloon aortic valvuloplasty; SAV: surgical aortic valvotomy / aortic valve repair; VSRR: valve sparing aortic root replacement; SAS: subvalvular AS; AVR: aortic valve replacement; MP-AVR: mechanical aortic valve replacement; BP- AVR: bioprosthetic aortic valve replacement; HG-AVR: homograft aortic valve replacement.*

**Supplement 15.**


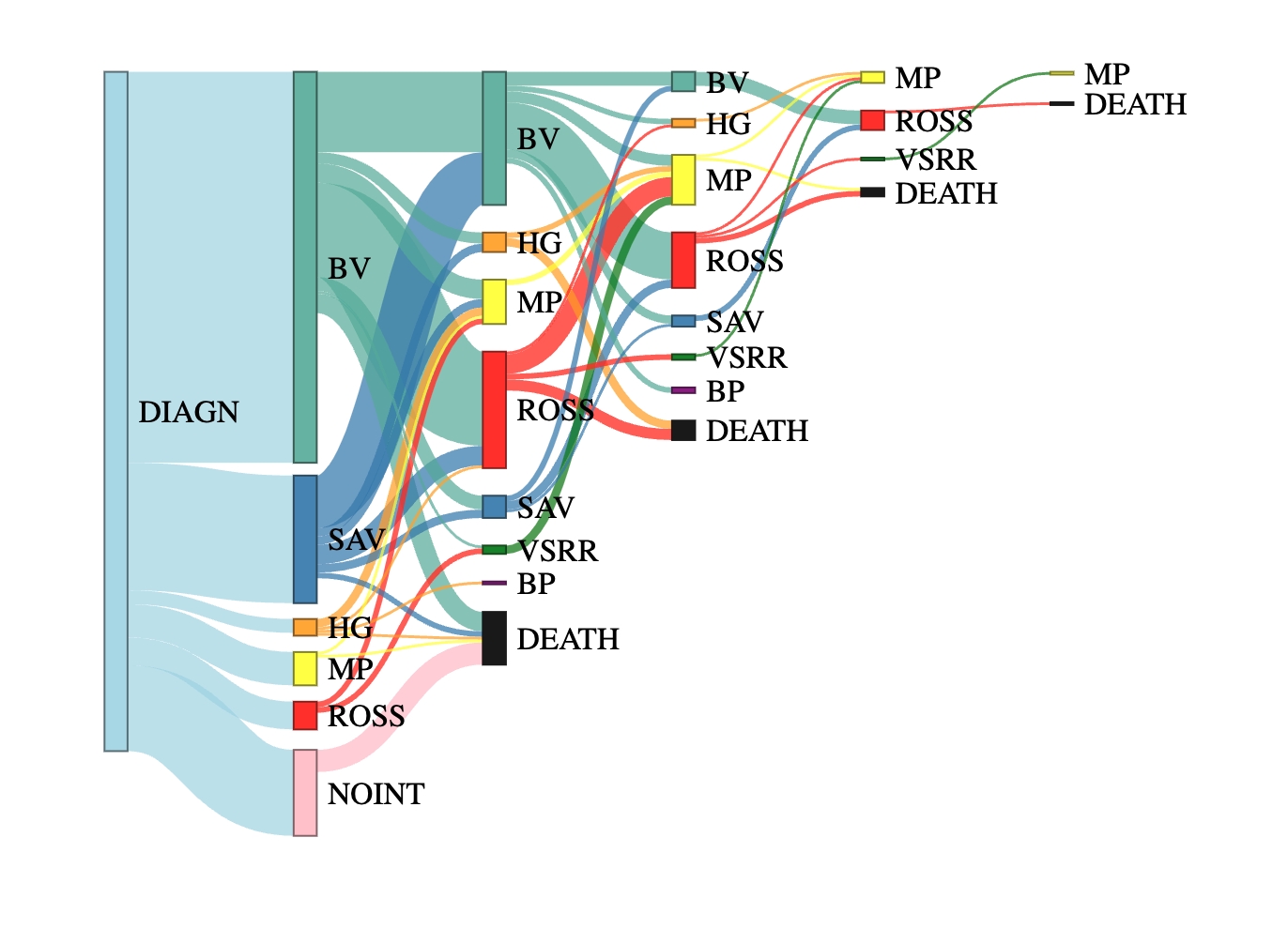


Sankey plot for all patients that underwent any intervention for their severe valvular AS. Interventions are colour coded. The width of the bar represents the number of patients in that intervention group. BV: Balloon aortic valvuloplasty; SAV: Surgical aortic valvotomy / aortic valve repair; HG-AVR: Homograft aortic valve replacement; MP-AVR: Mechanical aortic valve replacement; BP-AVR: Bioprosthetic aortic valve replacement; VSRR: Valve sparing aortic root replacement.

**Supplement 16.**


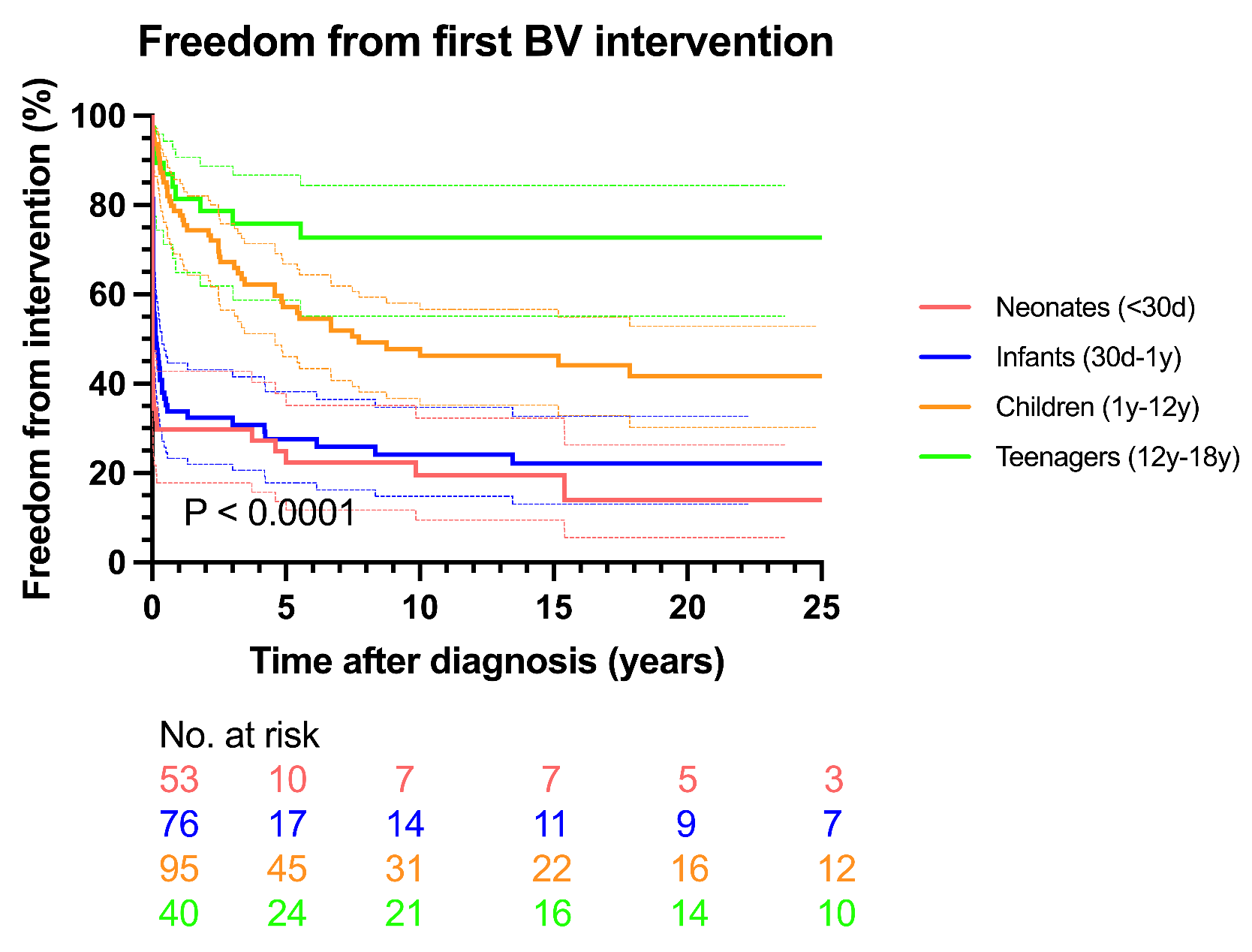


**Supplement 17.**

**
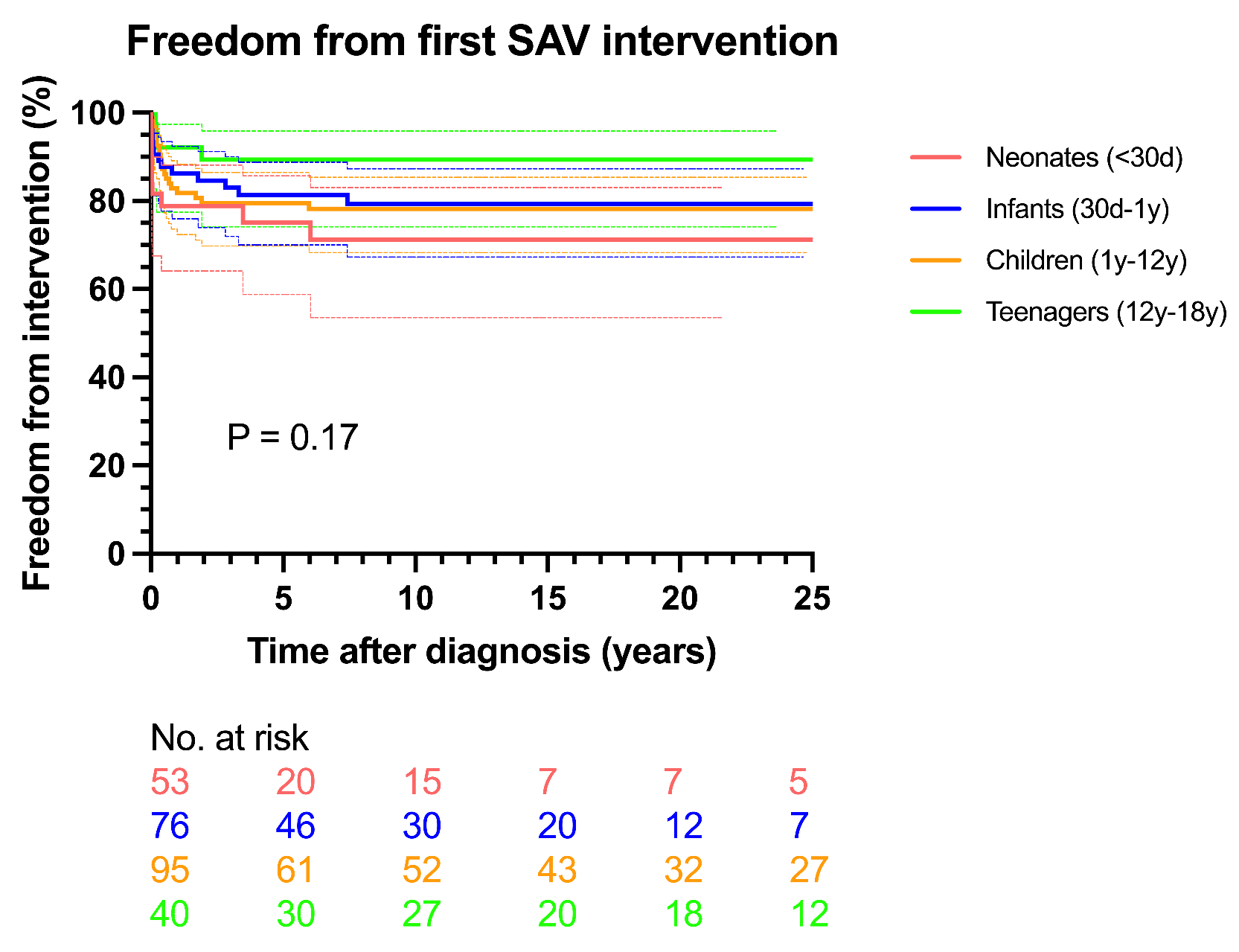
**

**Supplement 18.**

**
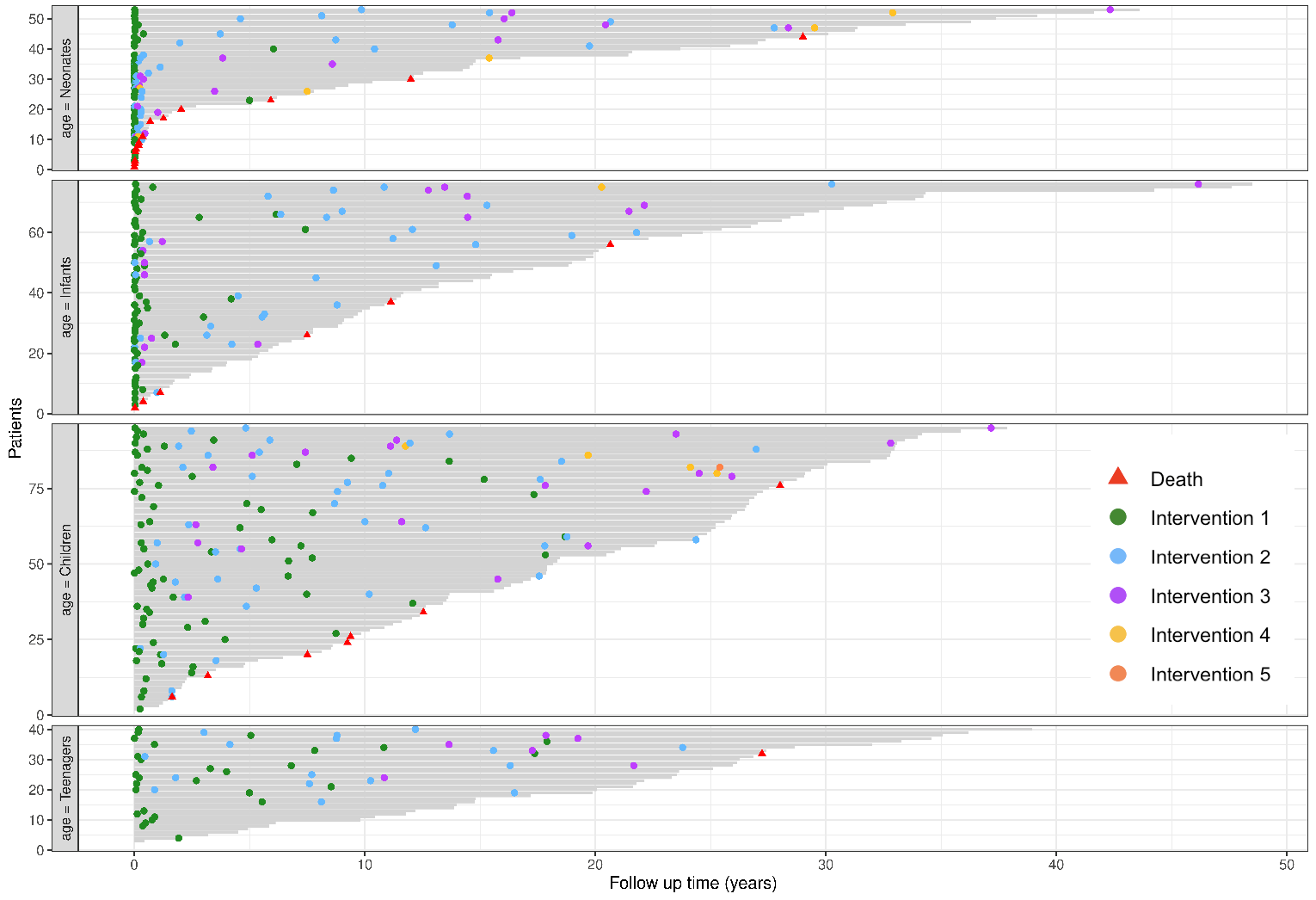
**

**
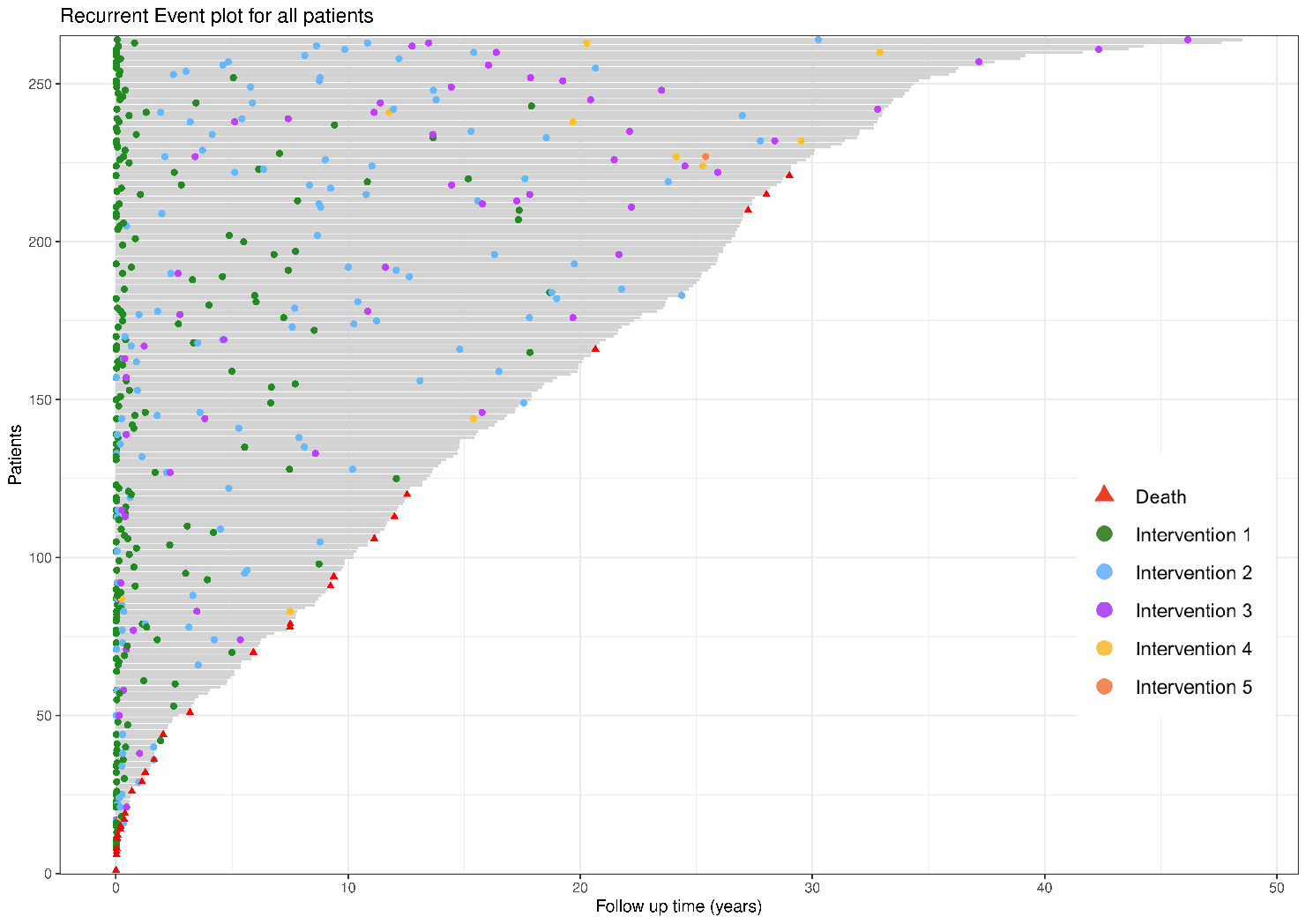
Supplement 19.**


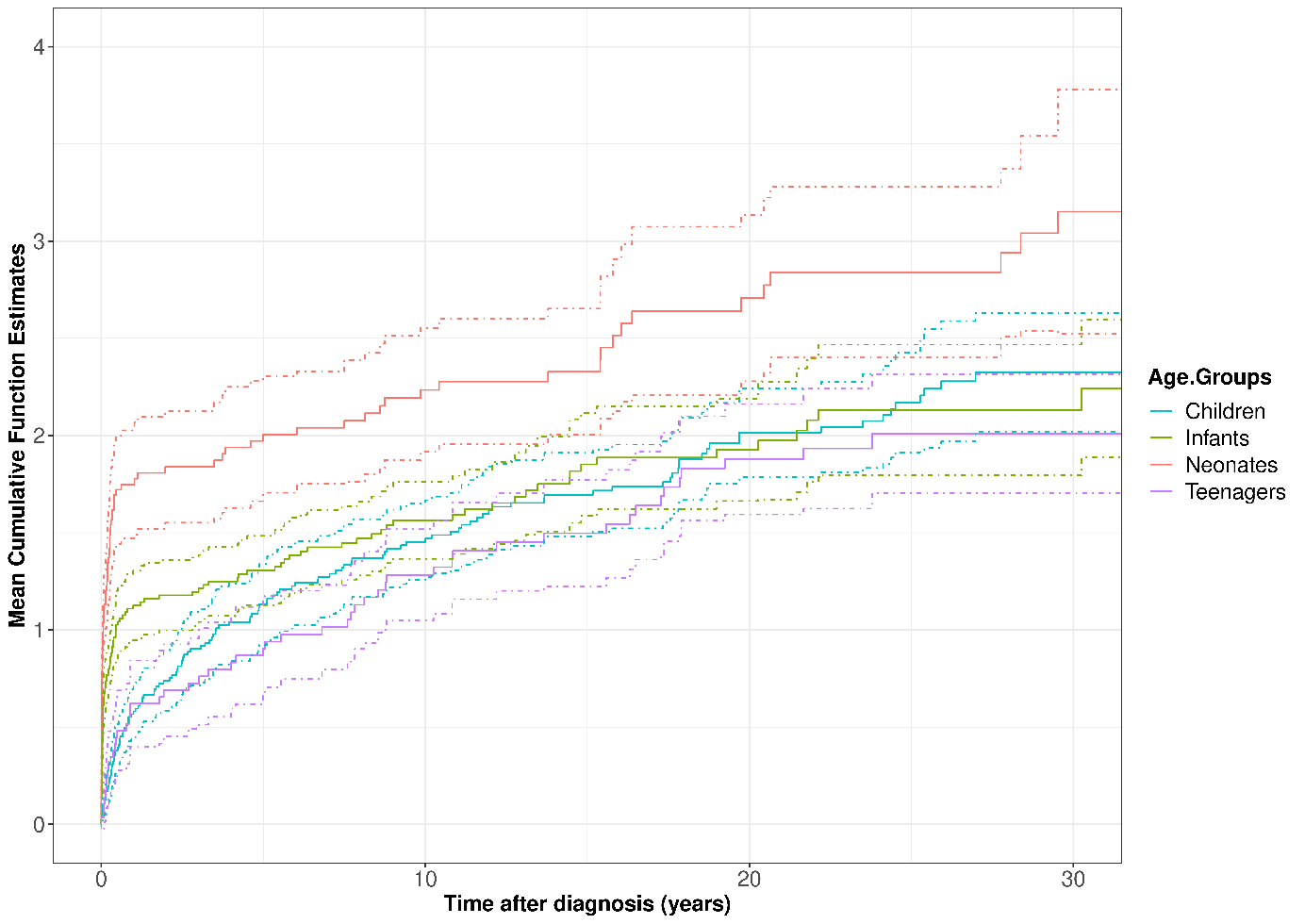
**Supplement 20.**

**Supplement 21.**

1. Jet velocity


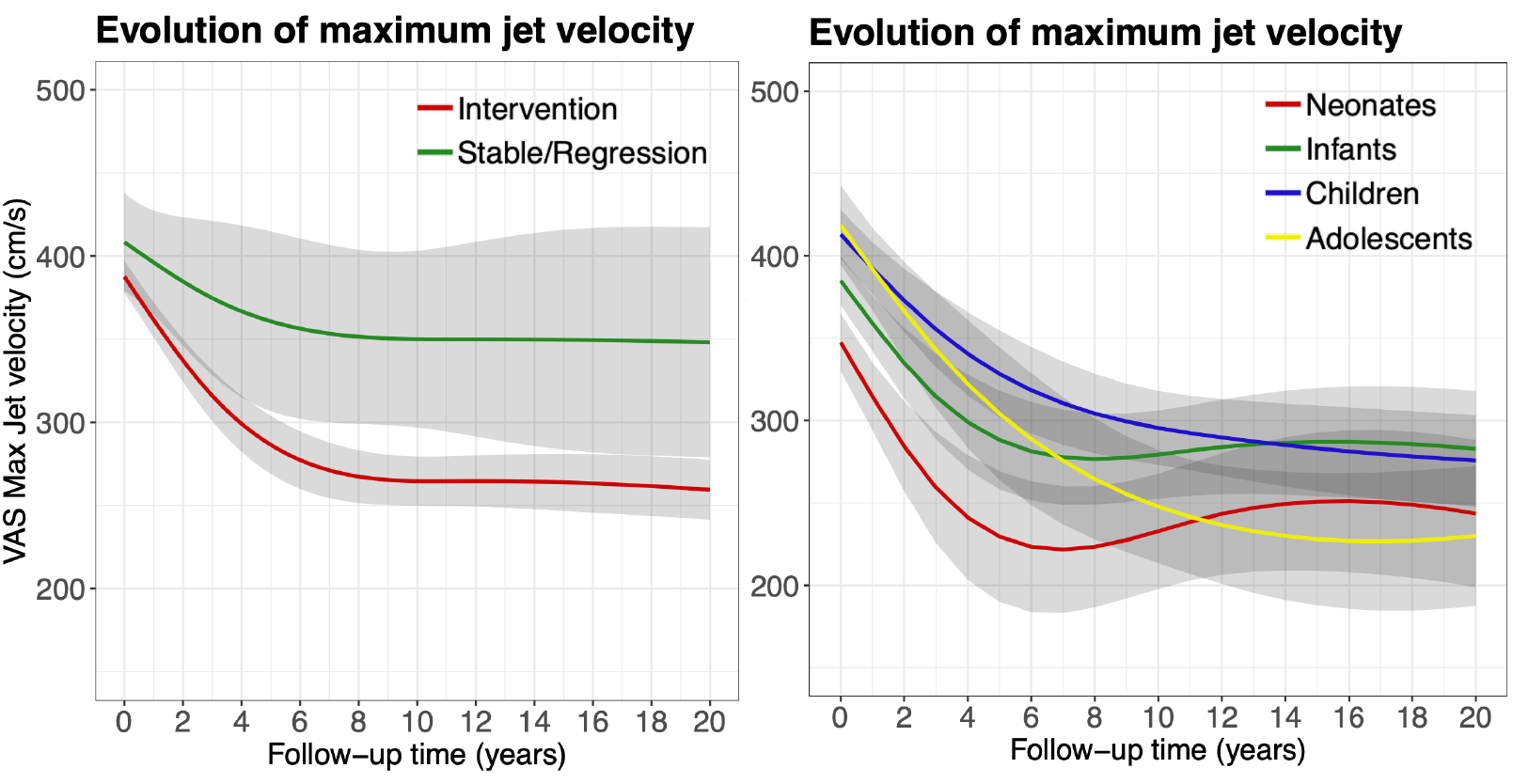


**B.** Fractional shortening


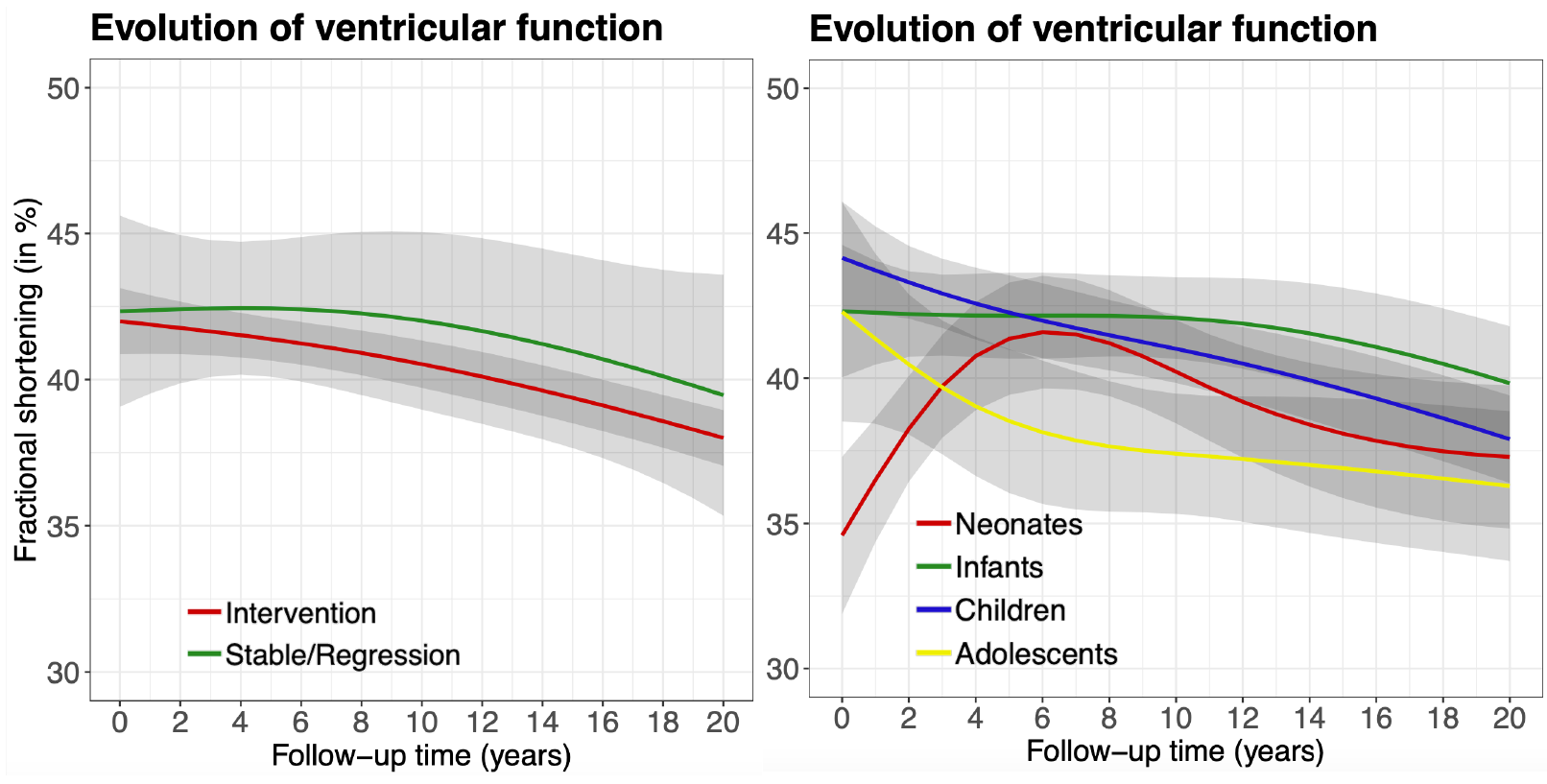


**Online** **References**

1. Ross RD. The Ross classification for heart failure in children after 25 years: a review and an age-stratified revision. Pediatric cardiology. 2012;33:1295-300.

2. Rubin DB. Multiple Imputation for Nonresponse in Surveys: Wiley; 1987. 258 p.
